# Diversity of Microbial Communities, PAHs, and Metals in Road and Leaf Dust of Functional Zones of Moscow and Murmansk

**DOI:** 10.3390/microorganisms11020526

**Published:** 2023-02-18

**Authors:** Anna A. Vetrova, Olesya I. Sazonova, Anastasia A. Ivanova, Rostislav A. Streletskii, Dmitriy A. Sarzhanov, Maria V. Korneykova, Andrey I. Novikov, Viacheslav I. Vasenev, Kristina V. Ivashchenko, Marina V. Slukovskaya, Olga Gavrichkova

**Affiliations:** 1 Federal Research Center “Pushchino Scientific Center for Biological Research of the Russian Academy of Sciences”, 142290 Pushchino, Russia; 2 Faculty of Soil Science, Laboratory of Ecological Soil Science, Lomonosov Moscow State University, 119991 Moscow, Russia; 3 Agrarian and Technological Institute, Peoples’ Friendship University of Russia (RUDN University), 117198 Moscow, Russia; 4 Institute of North Industrial Ecology Problems Subdivision of the Federal Research Center “Kola Science Centre of Russian Academy of Science”, 184209 Apatity, Russia; 5 I.V. Tananaev Institute of Chemistry and Technology of Rare Elements and Mineral Raw Materials, Kola Science Centre, Russian Academy of Sciences, 184209 Apatity, Russia; 6 Soil Geography and Landscape Group, Wageningen University, 6707 Wageningen, The Netherlands; 7 Laboratory of Nature-Inspired Technologies and Environmental Safety of the Arctic Region, Kola Science Centre, Russian Academy of Sciences, 184209 Apatity, Russia; 8 Research Institute on Terrestrial Ecosystems, National Research Council, 05010 Porano, Italy

**Keywords:** bacterial and fungal communities, PAH, metal, leaf dust, road dust, functional zones of city

## Abstract

The impact of geographical factors, functional zoning, and biotope type on the diversity of microbial communities and chemical components in the dust of urban ecosystems was studied. Comprehensive analyses of bacterial and fungal communities, polycyclic aromatic hydrocarbons (PAHs), and metals in road and leaf dust in three urban zones of Murmansk and Moscow with contrasting anthropogenic load were conducted. We found that the structure of bacterial communities affected the functional zoning of the city, biotope type, and geographical components. Fungal communities were instead impacted only by biotope type. Our findings revealed that the structure of fungal communities was mostly impacted by PAHs whereas bacterial communities were sensitive to metals. Bacteria of the genus *Sphingomonas* in road and leaf dust as indicators of the ecological state of the urban ecosystems were proposed.

## 1. Introduction

Urban dust is a complex heterogeneous system consisting of natural and technogenic particles [1,2]. In recent years, the number of studies exploring the microbial and chemical composition of dust has increased [3]. Urban dust is a significant indicator of the urban ecosystem, likely because dust is one of the main pollutants of the air in urban environments, consisting of big, fine, and ultrafine particles with a negative impact not only on the environment [4] but also on human health [5]. 

Dust’s harmful effects are triggered by a wide range of potentially toxic components: pathogenic microorganisms, PAHs, metals, soot, and other pollutants. Metals and PAHs are the primary anthropogenic pollutants [6]. Sources of environmental pollution by PAHs and metals are the burning of fossil fuels for energy production (thermal and electrical power), industrial processes (e.g., the metal industry or the cement/building industry), and the transport sector. Corrosion of car chassis and brake and tire abrasion generate high levels of benzo[a]pyrene [7] and benzo[g,h,i]perylene [8] as well as metals such as Cu, Ba, Fe, Zn, Cd, K, Mn, Sb, and Ti [9]. Materials used in road pavement construction can also be a source of PAHs emissions. The exhaust gases of car engines contain various PAHs such as naphthalene, phenanthrene, anthracene, chrysene, pyrene, fluorene, and benzo[a]pyrene [10]. The content of pyrene and fluorene was ten times higher than benzo[a]pyrene, which is an indicator of PAH pollution in the environment. 

PAHs and metals interact with the microbial communities in the environment. Findings showed that PAHs impact the beta- and deltaproteobacteria classes in materials used in urban landscaping [11]. Those authors suggested that the environmental microbiota is directly related to the commensal microbiota, the immune system, and human health. The increase in concentrations of heavy metals such as Cu [12], Cr, Mn, Pb, and Zn [13] negatively affected the alpha diversity of the microbial communities. Markowicz et al. [14], using phospholipid fatty acid (PLFA) and denaturing gradient gel electrophoresis (DGGE) methods, showed that heavy metals, not PAHs, were primarily responsible for the decrease in microbial activity, biodiversity, and differences in the microbial community structure. Meanwhile, dust microorganisms remain one of the less studied microbial components of the biosphere compared with the extensive amount of research on the biogeography of microorganisms in habitats such as soil and water [15,16]. Atmospheric dust transport is the main route by which both pollutants and microorganisms are spread [17]. Dust can sediment on such biotopes as sealed surfaces (asphalt, concrete pavement, etc.) and leaf surfaces and, under the influence of air masses, migrate between biotopes. The latter can lead to changes in the structure of the microbial communities of the biotopes [18,19,20,21].

In addition to anthropogenic factors, the structure and functioning of the biological components of the ecosystem, including the microbial community, are influenced by geographical and meteorological conditions [22,23]. Scholars have proposed that the distribution of bacteria and fungi in deposited dust at the continental scale correlates with regional environmental factors [23]. Such global studies of microbial community diversity distribution are scant and generally do not consider local scale factors such as functional urban zoning, traffic, vegetation, and pedestrians, which have been shown to play an important role in the formation of dust microbiomes on smaller spatial scales [19,24,25,26,27,28]. For example, the highest concentrations of pollutants associated with typical industrial pollutants are usually found in areas with a high traffic load [24]. Traffic areas through dust resuspension also increased the biodiversity of dust-associated communities in Rome [19]. Hence, the functional zoning of cities determines the degree of anthropogenic impact on ecosystems, which in turn can affect the structure and functioning of their biological components, plants, and microorganisms [25,26,27,28]. 

Researchers often limit their studies to the influence of only one factor, for example, the impact of one type of pollutant on the dust microbial communities of one biotope (phylloplane, sealed surfaces, or air) [29]. However, multiple interactive factors affect the dust microbial community structure, starting with the system from which the dust is collected to such environmental factors as temperature, pH, and humidity. Therefore, to disentangle natural (climatic zone) and anthropogenic (pollution) drivers, it seems appropriate to conduct a comprehensive study comparing the effects of different chemical pollutants on microbial communities in cities located in different geographical regions, considering the functional zoning of cities. The goal of this study was to determine the factors that have the greatest impact on the structural diversity of microbial communities of dust from sealed surfaces and leaves in three functionally different urban zones of Murmansk and Moscow with contrasting anthropogenic load. To increase the strength of the conclusions, we developed our experimental design to exclude the impact of instantaneous temperature differences and seasonal factors as well as the type of plant and its phenological stage of growth in the two cities.

## 2. Materials and Methods

### 2.1. Site Description and Sampling Procedures

The sampling campaign was conducted in the summer of 2021 in two big industrial cities of the Russian Federation located in the subarctic (Murmansk) and temperate continental (Moscow) climatic zones. The meteorological characteristics of the two cities and the conditions preceding the sampling are described in detail in [30,31]. Sampling in Moscow was carried out earlier than in Murmansk with the aim to ensure a similar seasonality and phenological stage of growth of selected tree species. 

In each city, three functional zones distinguished by the level of the anthropogenic load were selected for sampling: traffic zone, residential zone, and recreational zone. Data from local air quality monitoring stations were used to find suitable sites in Moscow [32]. For Murmansk, the assessment was based on the traffic load counts and visual assessment of the sites. The coordinates of the sampling points were as follows: the traffic zone, 68.960117 N, 33.064084 E (Murmansk) and 55.738328 N, 37.620061 E (Moscow); the residential zone, 68.978944 N, 33.093556 E (Murmansk) and 55.651983 N, 37.499363 E (Moscow); the recreational zone, 68.941098 N, 33.119497 E (Murmansk) and 55.833000 N, 37.549794 E (Moscow).

Dust was collected from two biological matrices (hereafter called “biotopes”): (1) leaves of *Betula pubescens* Ehrh. (hereafter called “leaf dust”) and (2) dust from the sealed road surfaces (hereafter called “road dust”). Three, mature, healthy birch trees similar in phenological development and state were selected in each zone and city. The sampling considered the entire circumference of the crown at a height of 1.5–2.5 m. In total, 150 leaves were collected from each birch and stored at 4 °C in sterile plastic bags until further analysis. Thus, 450 leaves were collected in each functional area. Three sites for road dust collection with an area of 1 m^2^ were located near the selected trees. Dust was swept with a sterile brush and collected in 50-mL tubes. Samples were stored at 4 °C. The obtained dust samples were put through a sterile sieve to obtain a dust fraction lower than 100 μm.

### 2.2. DNA Isolation from the Samples and 16S/ITS Metabarcoding

*Leaf dust.* In total, 60–70 leaves collected from three birch trees in each individual urban zone (surface area of ca. 300–350 cm^2^ for each tree; total area of about 900–1000 cm^2^ for each zone) were pooled together, placed in a sterile flask with 300 mL of sterile saline solution (8.5 g L−1), and thoroughly mixed on the shaker at 200 rpm for 15 min. Large impurities were removed from the obtained suspensions using a 100-μm mesh size grid. To collect the microorganisms, the suspension was further filtered through Nalgene Rapid-Flow with a 0.22 PES membrane (Thermo Fisher Scientific, Waltham, MA, USA). The membrane was cut with sterile scissors and transferred to the Power Bead Pro Tube (QIAGEN, Hilden, Germany). DNeasyPowerSoil Pro Kit (QIAGEN, Hilden, Germany) was used for DNA extraction from leaf dust according to the manufacturer’s protocol. The DNA was eluted with 50 μL of elution buffer.

*Road dust.* Road dust of less than 100 μm in size was thoroughly mixed. DNA extraction was performed from 250 mg of the pooled sample using the DNeasy PowerSoil Kit (Qiagen) according to the manufacturer’s protocol. The DNA was eluted with 50 μL of elution buffer.

*Metabarcoding.* Qubit 2.0 fluorometer (Invitrogen/Life Technologies, Carlsbad, CA, USA) was used to determine the amount of DNA. Universal primer pairs were used for polymerase chain reaction (PCR): _ forward: 341F 5′-CCTACGGGGNGGGGCWGCAG-3′ and reverse: 805R 5′-GACTACHVGGGTATCTAATCC-3′ (hypervariable V3–V4 regions of the bacterial 16S rRNA gene) [33]; forward: ITS1 5′-TCCGTAGGTGAACCTGCGG-3′ and reverse: ITS4 5′-TCCTCCGCTTGATATGC-3′ (region ITS1-5.8S-ITS2 of the fungal rRNA gene) [34]. The amplicon libraries for bacteria and fungi were prepared separately. Sequentia Biotech SL (Barcelona, Spain) performed the PCR, the library preparations for the next-generation sequencing, and the Illumina MiSeq sequencing (paired end, 2 × 300 cycles) of the bacterial 16S rRNA genes and fungal ITS region.

### 2.3. Bioinformatic Analysis

Adapter sequences were removed by quality trimming of unprocessed read sequences with Trimmomatic v0.32360. The FastQC toolkit (Babraham Bioinformatics, Cambridge, UK) was used to check sequence quality. The software GAIA (version 2.02, Sequentia Biotech, Spain) [35] was used for bioinformatic processing of trimmed raw sequences. The NCBI “nr” database was used for this analysis. All high-quality reads were rarefied to get even depths of minimal library and binned into operational taxonomic units (OTUs). OTUs at each taxonomic level (species, genus, family, order, class, and phylum) were obtained based on sequence similarity (97% identity) for each sample. 

### 2.4. Analyses of Chemical Components

#### 2.4.1. Analyses of PAH

*Leaf dust.* From the total number of collected birch leaves in each individual urban zone (surface area of ca. 250 cm^2^ for each tree), 30 leaves were selected and placed in a sterile flask with 30 mL of deionized water and thoroughly mixed on the shaker at 200 rpm for 15 min. The leaves were scanned, and their area was determined. The PAHs of 10 mL of methylene chloride were extracted from the obtained solution. The PAHs were determined by high performance liquid chromatography (HPLC) using an Agilent 1260 system (USA) with the fluorescence detector. We evaluated PAHs from the List of Priority Pollutants [36]: acenaphthene, anthracene, benzo[a]anthracene, benzo[b]fluoranthene, benzo[k]fluoranthene, benzo[a]pyrene, benzo[g,h,i]perylene, chrysene, dibenzo[a,h]anthracene, fluoranthene, fluorene, phenanthrene, and pyrene. The minimum detection limit for PAHs was 0.05 µg/kg. The recovery rate was 90%–97%. The PAH Calibration Mix (Merck, Germany) was used as a standard. The components were quantified by an absolute calibration curve method.

*Road dust.* An amount of 2 g of the sieved dust was taken from each of three replicate dust samples. The PAHs were extracted from these samples of road dust by 50 mL of methylene chloride and separated using the HPLC method that is described in the previous paragraph.

#### 2.4.2. Analyses of Metals

*Leaf dust.* From the total number of collected birch leaves in each individual urban zone (surface area of ca. 500 cm^2^ for each tree), 60 leaves were selected, placed in a sterile flask with 50 mL of deionized water, and thoroughly mixed on the shaker at 200 rpm for 15 min. The area of leaves was determined. Coarse impurities were removed from the obtained solutions by filtration through a 100-μm mesh. The filtrate was then placed in an oven at 65 °C for complete evaporation of the water. Evaporated deionized water was used as a control. The concentration of different metals (Ag, Al, As, Au, B, Ba, Be, Bi, Ca, Cd, Ce, Co, Cs, Cr, Cu, Dy, Eu, Er, Fe, In, Ir, Hf, Ho, Ga, Ge, Gd, K, La, Li, Lu, Mg, Mn, Mo, Na, Nb, Nd, Ni, Pb, Pd, Pt, Pr, Rb, Re, Ru, Rh, Ta, Tb, Te, Ti, Tm, Th, Tl, Sb, Sc, Se, Si, Sm, Sn, Sr, U, V, Y, Yb, W, Zn, Zr) in the sample was measured using the ELAN 9000 DRC-e (Perkin Elmer, Waltham, MA, USA) mass spectrometer with inductively coupled plasma (ICP MS).

*Road dust.* An amount of 1 g of the sieved dust was taken from each of three replicate dust samples. The metals were separated by a mass spectrometer with inductively coupled plasma that is described in the previous paragraph.

### 2.5. Statistical Analysis

The descriptive statistics and visualization of metals and PAHs content and relative abundance of bacterial and fungal communities in the samples were carried out using Microsoft Office Excel 2019. The data were treated to obtain the mean and standard error. 

Statistical analyses of microbial communities (such as bacterial and fungal) were performed by the MicrobiomeAnalyst program [37] using default parameters. The R v4.2.1 statistical programming language (R Development Core Team 2022, Vienna, Austria) with the associated packages was used to statistically analyze and visualize the alpha diversity of microbial communities (the Chao1 richness and Shannon) across all types of samples. Beta diversity was estimated on the computation of the pairwise Bray–Curtis index dissimilarity matrix at the classes level and at the species level in the bacterial and fungal communities. Beta diversity at the species level was estimated by the nonmetric multidimensional scaling (NMDS) ordination of variance stabilized counts of taxa for all analyzed samples, compared using Bray–Curtis dissimilarity. Dendrograms of bacterial/fungal community relationships were constructed to estimate beta diversity at the classes level. Permutation analysis of variance (PERMANOVA) and corresponding R-squared and p-values were calculated. Statistical differences in mean alpha diversity values between biotopes and functional zones were investigated with the post-hoc Tukey’s HSD tests after providing significance within the factorial ANOVA analysis.

The online resource Venny [38] was used to obtain the Venn diagram. Venn diagrams demonstrated the qualitative relationship between species present in the bacterial and fungal dust communities of different biotopes, taking into account the functional zoning of cities. As input data for constructing the Venn diagram, we did not use the number of OTU of each identified species but the presence of this species itself in the microbial community.

The heat maps and hierarchical clustering were performed in Morpheus [39] using the one minus Pearson correlation. The hierarchical clustering recursively merged the objects based on their pair-wise distance. The heat maps were constructed to study the correlations between the bacteria and fungi at the species level (the normalized relative abundance) and sites/biotopes. Other heat maps were constructed to explore the correlations between the chemical parameters (the normalized relative abundance of PAH and metal concentrations) and sites/biotopes.

Redundancy analysis (RDA) was performed in XLSTAT using R-package 4.2.1. to detect the relationship between the bacterial/fungal phyla, the studied PAHs and metal, and the biotopes of the functional zones of the cities. We used a pairwise correlation matrix to look for the main components based on the relationship of the properties of the two cities’ functional zones (the normalized PAH/metal concentration and microbial phyla) using the Pearson correlation coefficient.

Principal component analysis (PCA) was performed using the ClustVis Internet resource [40] to compare of the PAH/metal content in different zones/biotopes of the two cities.

## 3. Results

### 3.1. Diversity of Bacterial and Fungal Communities

We detected a total of 3,661,626 bacterial 16S-amplicon sequences and 2,046,128 fungal ITS-amplicon sequences in two biotopes of three functional zones in Murmansk and Moscow. After quality filtering, our 16S and ITS datasets comprised 3,654,254 and 1,649,708 sequences, which were clustered into 6343 bacterial species-level OTUs and 4340 fungal species-level OTUs. 

#### 3.1.1. Chao1 Richness and Shannon Diversity Indices 

**Figure 1:**
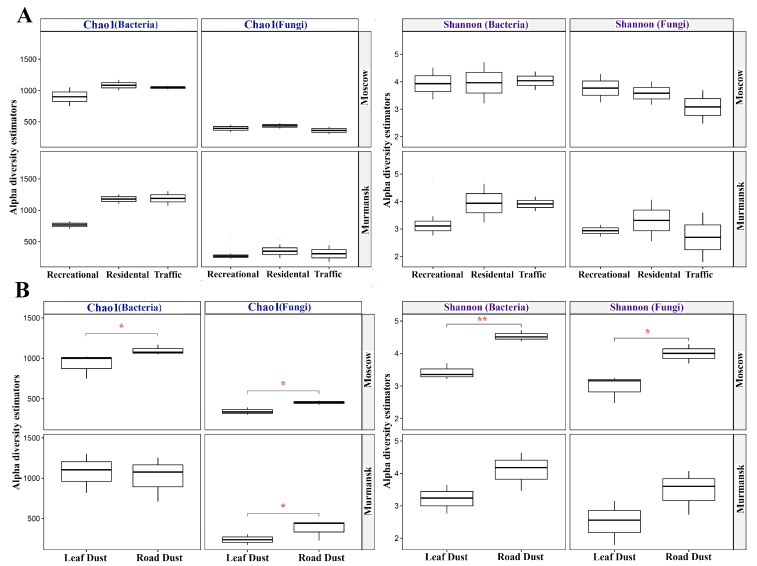
Comparisons of Chao1 richness and Shannon diversity of bacterial and fungal communities between functional zones (**A**) and biotopes (**B**) of Moscow and Murmansk. The horizontal lines show significance levels between groups (* *p* < 0.05, ** *p* < 0.01).

Richness and diversity indices of bacterial and fungal communities were calculated from the sequencing data (Figure 1). The Chao1 richness index was lower for bacterial communities in recreational zones of both cities. The Shannon index of bacterial communities in the recreational zone of Murmansk was also lower than in other functional zones. No similar effect was observed for Moscow samples. For fungal communities, we observed a decrease in the Shannon index from the recreational zone to the traffic zone only for samples collected in Moscow. In the fungal community of Murmansk, Chao1 richness was lower in the recreational zone than in other functional zones. As for the biotope effect, the indices of Chao1 richness and Shannon diversity in the road dust were nearly always significantly higher than in the leaf dust for fungal and bacterial communities in the two cities.

#### 3.1.2. Taxonomic Composition of Bacterial and Fungal Communities 

**Figure 2:**
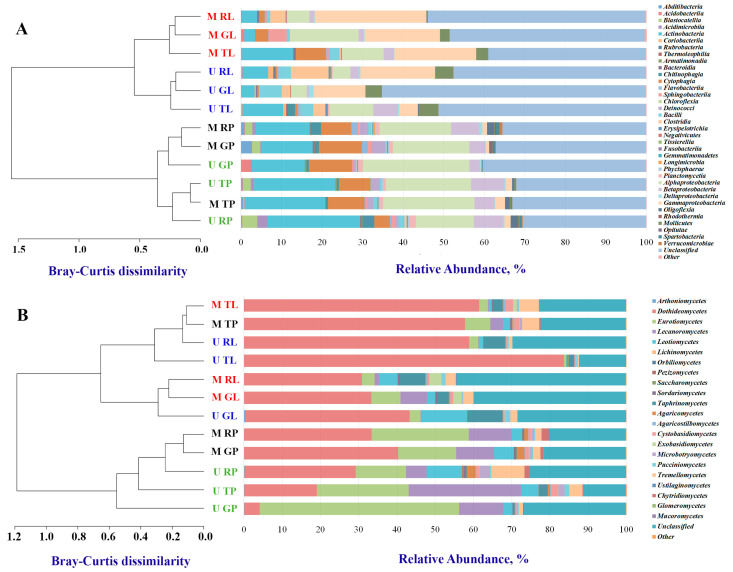
Distribution of the most abundant bacterial (**A**) and fungal (**B**) classes (read frequency > 0.1%) identified in functional zones and biotopes of Moscow and Murmansk. Unclassified phyla and phyla with relative abundance ≤0.1% were considered as “Unclassified” and “Other”, respectively. Clustering analysis of the class-level communities based on the computation of the pairwise Bray–Curtis dissimilarity matrix is also reported. The first letter in the abbreviation refers to the city: M–Moscow, U–Murmansk; the second letter refers to the functional zone: T–traffic, R–residential, G–recreational; and the last letter, to the biotope: L–leaf, P–paved surface.

Bacterial samples ranged from 85,465 to 118,472 reads. We detected a total of 42 identified phyla. Ten to 18 of the bacteria phyla had a relative abundance above 0.1%, depending on the biotope, the functional zone, and the city. Phylum *Proteobacteria* was the most dominant (range 23.2%–38.6%), followed by *Actinobacteria* (3.0%–26.5%), *Cyanobacteria* (2.0%–17.5%) and *Bacteroidetes* (1.0%–15.3%). Within the *Proteobacteria*, most phylotypes were represented by *Alphaproteobacteria* (3.8%–26.3%), *Betaproteobacteria* (0.5%–8.2%), and *Gammaproteobacteria* (0.2%–27.6%). It should be noted that *Alphaproteobacteria* (14.3%–26.3%) and *Betaproteobacteria* (2.5%–8.2%) prevailed in the biotope of road dust, and *Gammaproteobacteria* (4.6%–27.6%)–in leaf dust. We detected the highest relative abundance of *Gammaproteobacteria* in the residential zone in leaf dust. *Actinobacteria* were mostly represented by the classes *Actinobacteria* (2.7%–22.9%) and *Bacteroidetes* by *Cytophagia* (0.3%–10.7%) (Figure 2A, Table S1, Figure S1A). The last two classes prevailed in road dust. We observed an increase in the relative abundance of *Actinobacteria* in the recreational zone toward the traffic zone.

Fungal samples varied from 7511 to 89,275 reads. Six out of seven fungi phyla had a relative abundance above 0.1%. Total *Ascomycota* accounted for more than 60.7%–92.4% of all sequencing reads. There were 22 classes of fungi with a relative abundance above 0.1%. Total *Dothideomycetes* (p_ *Ascomycota*) accounted for 3.9%–83.6%, *Eurotiomycetes* (p_ *Ascomycota*) accounted for 0.5%–52.2%, and *Lecanoromycetes* (p_ *Ascomycota*) accounted for 0.1%–29.4%. The phylum *Basidiomycota* was better represented by *Tremellomycetes* (0.3%–8.7%), depending on the biotope, the functional zone, and the city (Figure 2B, Table S2, Figure S1B). Classes *Eurotiomycetes* and *Lecanoromycetes* prevailed in the road dust. Class *Taphrinomycetes* dominated in the leaf dust. Class *Dothideomycetes* was dominant in the traffic zone of the leaf dust of both cities. It is important to note that the data obtained for the bacterial and fungal communities were similar between the two cities in terms of dominant taxa.

#### 3.1.3. Similarity of Bacterial and Fungal Communities in Studied Sites

A dendrogram of the bacterial communities’ similarities constructed at the class level demonstrated that the communities of the two biotopes differed. Bacterial communities of the leaf dust clustered separately between the two cities (Figure 2A). For fungal communities (Figure 2B), we detected no clear clustering between biotopes or cities. Bacterial and fungal communities of the recreational and residential zones of Moscow shared similarities.

A Bray–Curtis distance matrix was also analyzed with NMDS to explore the beta diversity of all samples at the species level. The NMDS plot indicated that the biotopes had a greater impact on the bacterial and fungal communities of the studied sites than the functional zones of the cities and the cities’ climatic belt (Figure S2). We suggest that the impact of different climatic belts was significant only for fungal communities (*p*-value = 0.0653). 

All the possible relationships among the communities of different biotopes, functional zones, and cities are schematically represented in the Venn diagrams (Figure S3, Figure S4). The number of species between the two biotopes was comparable in Moscow and Murmansk. At the same time, the number of species in Murmansk was 1.25 times higher than in Moscow for both biotopes. We identified 184 common elements among the OTUs of all the studied microbiomes. The following bacteria identified to species level were common to all studied samples: *Abditibacterium utsteinense, Blastocatella fastidiosa, Noviherbaspirillum suwonense, Aquihabitans daechungensis, Loriellopsis cavernicola, Sphingomonas echinoides,* and *Sphingomonas solaris* (data not shown). In Moscow, the number of similar bacterial species in leaf dust samples of functional zones compared with road dust was 1.5 times lower (Figure S3A). In Murmansk, no similar effect was found. These data were comparable (Figure S3B). We detected the lowest number of species, including unique species, in the recreational zone. Leaf dust contained the maximum number of similar species between the traffic zone and the residential zone, and the minimum number between the recreational zone and the residential zone. The maximum number of unique species was found in the traffic area in the leaf dust. The number of unique species in the recreational zone was comparable among all biotopes (Figure S3A,B). As shown in the Venn diagram (Figure S3C), the number of similar species between cities and biotopes was two times lower in the recreational zone than in other functional zones.

There were 51 common elements among the OTUs of all the studied microbiomes, with the following common fungal species: *Aureobasidium pullulans, Sydowia polyspora, Genolevuria tibetensis, Pseudopezicula betulae, Taphrina nana, Vishniacozyma tephrensis, Venturia Helvetica, Prosthemium asterosporum, Filobasidium wieringae, Endoconidioma euphorbiae, Buckleyzyma aurantiaca, Xanthoria parietina*, and *Neocucurbitaria cava*. The smallest number of species, including unique species, was detected in the green zone of Moscow and in the dust from the asphalt of Murmansk (Figure S4A,B). As in the case of bacterial communities, fungal communities had the lowest number of total species in the recreational zone (Figure S4C). 

### 3.2. Chemical Composition of Leaf and Road Dust of the Cities’ Functional Zones

The qualitative and quantitative content of metals and PAHs was analyzed in the studied samples. Of the 66 metals studied, only nine showed some correlation with the degree of anthropogenic pressure (Figure S5). Rubidium content decreased in both biotopes of Moscow and Murmansk from the traffic zone to the recreational zone. Similarly, we found that only Mg and Al decreased in leaf dust and that B decreased in road dust. In addition, the content of Mg and Al in Moscow was significantly lower than in Murmansk. In Moscow, the content of Ni and Ca increased in road dust and decreased in leaf dust from the traffic zone to the recreational zone. In Murmansk, Ba concentration decreased in both biotopes in the same direction.

A decreasing trend in fluoranthene, fluorene + acenaphthene, naphthalene, and ben[k]fluoranthene content from the traffic to the recreational zone in both cities was detected only for leaf dust (Figure S6). For the road dust, a similar effect was observed for anthracene and pyrene. Benzo[a]antracene and benzo[a]pyrene decreased in the direction of lowering the anthropogenic load in Murmansk. For naphthalene in the leaf dust, we observed a decrease in its concentration in Moscow, and the opposite effect was detected in Murmansk.

**Figure 3:**
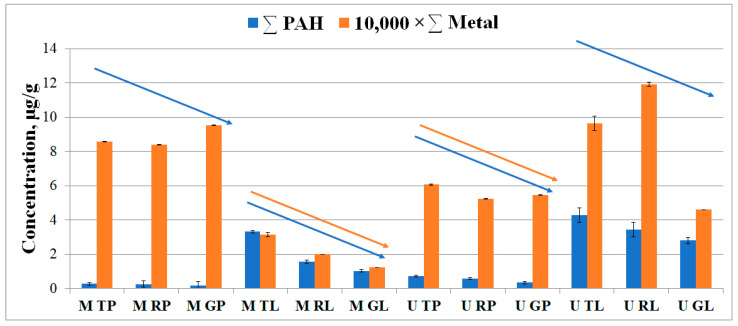
Total content of the PAHs (blue) and metals (orange) in biotopes of functional zones of Murmansk and Moscow. The first letter in the abbreviation refers to the city: M–Moscow, U–Murmansk; the second letter refers to the functional zone: T–traffic, R–residential, G–recreational; and the last letter refers to the biotope: L–leaf, P–paved surface. The data for metals were scaled down by a factor of 10,000 for visualization in the picture and compared to the dimensionality of the data for PAHs.

Our findings showed that the total metal content was significantly higher than the PAH content in the samples (Figure 3). There was a tendency for the total PAH content to decrease in the direction from the traffic zone to the recreational zone in both biotopes of Moscow and Murmansk. For the metal content, a similar effect was observed only for the leaf dust in Moscow and for the road dust in Murmansk. The qualitative and quantitative composition of PAHs and metals in different biotopes and functional zones of Moscow and Murmansk was compared using principal component analysis (PCA). PCA showed that the studied sites had more similarity in PAH content than in metals (Figure S7).

### 3.3. Correlation of the Studied Characteristics of Functional Zone Biotopes

We performed a Pearson correlation analysis to determine the probable relationship among bacterial and fungal communities, PAHs, and metals. Figure S8 shows the correlation analysis between the biotopes of the functional zones of Moscow and Murmansk and the relative representation of bacteria and fungi at the species level. Figure S9 shows the relationship between the biotopes of the functional zones of Moscow and Murmansk and the quantitative and qualitative composition of metals and PAHs. Metals were clustered in biotopes and cities, in contrast to PAHs and fungal and bacterial communities. All in all, the clustering of bacterial communities and PAHs was similar.

**Figure 4:**
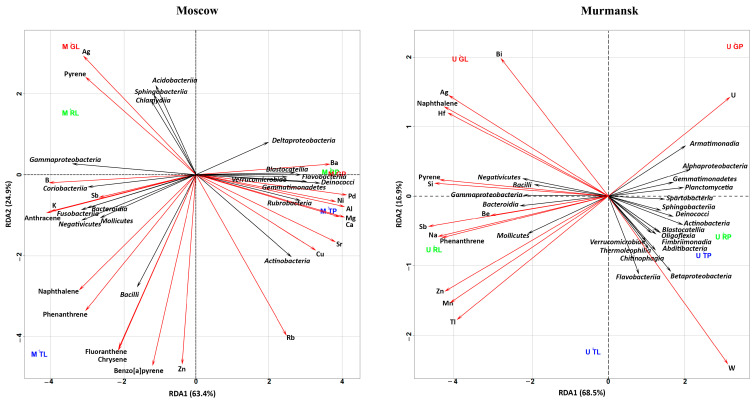
RDA ordination triplot showing the correlations of chemical pollutants (red arrows) and bacterial classes (black arrows) of the leaf dust and road dust from Moscow and Murmansk. The first letter in the abbreviation refers to the city: M–Moscow, U–Murmansk; the second letter refers to the functional zone: T–traffic, R–residential, G–recreational; and the last letter refers to the biotope: L–leaf, P–paved surface. The figures show only those classes of bacteria, metals, and PAHs that had a correlation with each other.

**Figure 5:**
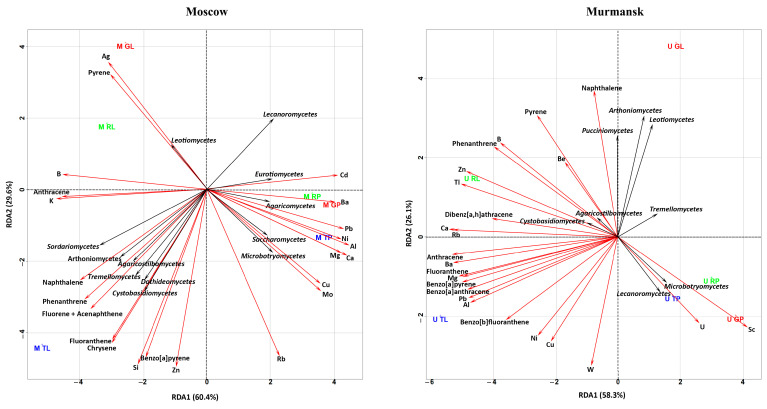
RDA ordination triplot showing the correlations of chemical pollutants (red arrows) and fungal classes (black arrows) of the leaf dust and road dust from Moscow and Murmansk. The first letter in the abbreviation refers to the city: M–Moscow, U–Murmansk; the second letter refers to the functional zone: T–traffic, R–residential, G–recreational; and the last letter refers to the biotope: L–leaf, Ppaved surface. The figures show only those classes of fungi, metals, and PAHs that had a correlation with each other.

Redundancy analysis (RDA) was used to illustrate the correlation between pollutant concentrations (metals, PAHs) and bacterial (Figure 4) and fungal (Figure 5) communities in different biotopes and cities. We conducted separate analyses for Moscow and Murmansk and included both biotopes. Bacterial and fungal communities were used at the class level.

#### 3.3.1. RDA of Bacterial Classes and Chemical Compositions

The first two axes described, together, 85.32% and 93.53% of the total variance for Murmansk and Moscow, respectively (Figure 4). The different biotopes of the two cities were grouped in opposite planes with respect to RDA 2. Most bacterial classes of Murmansk (7.8%–58.0% from total out content) except *Negativicutes, Bacilli, Gammaproteobacteria, Bacteroidia,* and *Mollicutes* (0.5%–26.3% from total OTU content) were negatively correlated with naphthalene, pyrene and phenanthrene, and metals (Si, Hf, Ag, Sb, Be, U, Na, Zn, and Mn). Bacterial classes (*Negativicutes, Bacilli, Gammaproteobacteria, Bacteroidia,* and *Mollicutes)* in positive correlation with the above chemicals were mostly represented in the leaf dust. Bacterial classes in negative correlation with the above chemicals were mostly represented in the road dust. *Oligoflexia, Blastocatellia, Fimbriimonadia, Abditibacteria, Verrucomicrobiae, Chitinophagia, Flavobacteriia, Betaproteobacteria,* and *Thermoleophilia* positively correlated with W content. U positively correlated with *Armatimonadia* and *Alphaproteobacteria* and negatively with *Mollicutes*. The RDA of the Moscow samples showed a positive correlation with *Acidobacteria, Sphingobacteria, Chlamydia, Gammaproteobacteria, Coriobacteria, Bacteroidia, Fusobacteria, Mollicutes, Negativicutes,* and *Bacilli* (1.4%–29.3% from total OTU content) bacteria classes mainly with PAH and some metals (Ag, Sb, and B). *Deltaproteobacteria, Blastocatellia, Flavobacteriia, Verrucomicrobiae, Deinococci, Gemmatimonadetes, Rubrobacteria,* and *Actinobacteria* (3.2%–22.4% from total OTU content) correlated positively mainly with metals (Ba, Pd, Ni, Al, Mg, Ca, Cu, Rb, and Sr) and negatively with PAHs. In general, for both cities, *Bacilli* were positively correlated with naphthalene and phenanthrene, and *Mollicutes* were positively correlated with phenanthrene. 

#### 3.3.2. RDA of Fungal Classes and Chemical Compositions

The fungal classes *Lecanoromycetes* and *Microbotryomycetes* positively correlated with U and Sc and negatively correlated with phenanthrene, pyrene, Zn, B, and Be in the Murmansk samples. *Tremellomycetes* negatively correlated with fluoranthene, benzo[a]anthracene, benzo[a]pyrene, benzo[b]fluoranthene, Mg, Pb, and Al. *Arthoniomycetes*, *Leotiomycetes*, *Agaricostilbomycetes*, *Cystobasidiomycetes*, and *Pucciniomycetes* were positively/negatively associated with metals and PAHs. It should be noted that the relative abundance of the above classes in the fungal community of Murmansk varied between 1.5% and 42.5%. 

The *Arthoniomycetes*, *Sordariomycetes*, *Agaricostilbomycetes*, *Dothideomycetes*, and *Tremellomycetes* classes of fungal communities from Moscow dust were positively correlated with naphthalene, phenanthrene, and fluorene + acenaphthene. *Cystobasidiomycetes* was positively correlated with fluoranthene, chrysene, benzo[a]pyrene, and Si. *Lecanoromycetes* were negatively correlated with the above-mentioned PAHs and Si. *Leotiomycetes* were positively correlated with Ag and pyrene and negatively correlated with Cu and Mo. *Microbotryomycetes* and *Saccharomycetes* showed inverse dependence compared with *Leotiomycetes*. *Eurotiomycetes* and *Agaricomycetes* were negatively correlated with B, K, and anthracene. *Eurotiomycetes* were positively correlated with Cd and Ba. *Agaricomycetes* were positively correlated with Ba, Pb, Ni, Al, Mg, and Ca. The classes *Lecanoromycetes*, *Leotiomycetes*, *Eurotiomycetes*, and *Dothideomycetes* were mostly represented in the fungal communities of Moscow dust. *Microbotryomycetes*, *Arthoniomycetes*, *Leotiomycetes*, *Agaricostilbomycetes,* and *Cystobasidiomycetes* showed similar correlations with respective PAHs and metals in both cities.

## 4. Discussion

Different environmental factors execute their pressure on the dust microbial community structure. Such environmental factors may include geographical location, meteorological conditions, seasonality, ultraviolet and chemical impacts (metals, PAHs, etc.), and type of biotope. It should be noted that even within the same urban system, there may be differences in the factors that impact it. For this reason, the urban ecosystem in the current study was divided into functional zones of the city.

We attempted to estimate the impact of various chemical pollutants on the microbial communities of road and leaf dust in cities located in different geographic regions, considering the functional zoning of the cities. It is known that the geographical locations of sample collection sites affect the microbial community structure in deposited dust and in the open air and that this impact is related to the climatic factors [41]. In our study, we excluded the short-term impact of the climatic drivers on microbial communities from the setup by shifting the sampling between Moscow and Murmansk in time to achieve similar temperatures and similar plant phenological development states. Climatic factors are limited to long-term effects of different climatic belts. In the present work, we did not address the seasonal variability of microbial community patterns. Including the season as a factor will probably add some additional patterns. Such studies are important from the practical point of view to identify certain taxa that can be used as universal bioindicators of the state and pollution of the environment. To do so, a thorough quantitative and qualitative analysis of anthropogenic pollutants (e.g., metals and PAHs) is of crucial importance.

### 4.1. Content of PAHs and Metals in Urban Dust Samples

The key components that have a negative impact on the environment are various chemical pollutants, such as metals and PAHs. In our study, the greater diversity of metals in the road dust compared with the leaf dust is likely due to the greater accumulative properties of the road surface [42]. However, we detected higher concentrations of some metals, and PAHs in the studied areas were detected in leaf dust. Road dust accumulates for a long period, concentrating the pollution washed out from precipitation from other surfaces. Hence, it is expected that road dust would have a low sensitivity to short-term changes in the air quality. Leaves instead concentrate the pollution between the rain events and are more sensitive to short-term pollution level variations. 

In Murmansk, the content of W, Cs, Ba, V, Zn, Rb, Co, La, Gd, U, and Al was higher than in Moscow. This may be explained by the presence of heavy industry in the Murmansk region. The main sources of anthropogenic emissions into the atmosphere of Murmansk are emissions from thermal power plants and household heating based on fuel oil. The main pollutants from thermal power plants are sulfur dioxide, nitrogen oxides, carbon monoxide, formaldehyde, and benzo[a]pyrene. Together with gaseous and liquid substances, fuel oil ash and products of the incomplete burning of fuel, which include heavy metals V, Ni, Cr, Pb, Fe, and Sn, enter the atmospheric air. Technogenic compounds of heavy metals and other pollutants coming from enterprises of the power unit are precipitated by wet and dry deposition on the various surfaces [43]. The functional zoning of cities impacts the total content of PAHs in the biotopes of both Moscow and Murmansk [31]. The absence of an anthropogenic gradient regarding most of the metals could be associated not only with the accumulation effect as a result of their technogenic nature but also with the presence of similar chemical elements in natural systems [6]. 

### 4.2. Factors Determining the Diversity of Microbial Communities

#### 4.2.1. Chao1 and Shannon Indices

The phyllosphere is a unique environment because of its structure, ecology, the accessibility of nutrients and humidity, and the impact of meteorological factors and solar radiation [41]. On the one hand, the leaves’ environment is relatively hostile to the microorganisms; on the other hand, the host plant provides the microorganisms with nutrients and shelter [44]. One can hypothesize that road dust is characterized by more extreme conditions in terms of microclimatic factors and nutrients supply, and that such conditions can limit the Chao1 richness and Shannon diversity of microbial communities. However, we found the opposite effect. Our results showed that the Chao1 richness and Shannon diversity of the leaf dust microbial community were lower than that of road dust (Figure 1). This is probably due to the presence of an additional factor impacting the structure of bacterial and fungal communities on the leaf surface. This additional factor is that the host plant itself is capable of a strict selection of the proper microbial pool by regulating the phylloplane environment (pH, nutrients availability, etc.). Some plant exudates, especially compounds of a phenolic nature, are capable of limiting the physiology of some microorganisms, including pathogenic microflora [45]. For example, studies have demonstrated that soil microbial communities are more varied than leaf microbial diversity [46]. 

In recent years, the microbial diversity of urban dust has been actively studied, especially in relation to its negative impact on the environment. The Chao1 and Shannon indices are often used to describe microbial diversity. Wuyts et al. [47] showed that when considering individual urban factors, Chao1 bacterial richness and the Shannon diversity index increased significantly with the distance of sites to the nearest recreational area, with no significant correlation with any other ecological factors. The results obtained in the present work are consistent with those data [19,45]. The functional zones of cities, or rather the anthropogenic gradient, also had no effect on the Chao1 richness of fungal communities, both in the present work and according to the study [19]. The Chao1 richness of the bacterial community was lower in the recreational zones of both cities, similar to Rome. Considering the median value, we suggest that the functional zoning of the cities influenced the alpha diversity of only the bacterial communities of both cities. Major pollution of residential and traffic zones promoted a shift in the taxonomic structure of the communities [3], likely contributing to the development of species that not only are resistant to pollution but also degrade pollutants and their oxidation products. 

The indices of Chao1 richness of fungal communities in Moscow and Murmansk were comparable to the same indices obtained for road dust and leaf dust in the samples from Rome [19]. Conversely, the indices of Chao1 of bacterial communities in Moscow, Murmansk, and Rome differed. Fungal communities are likely less sensitive to the geographical factors than bacterial communities. 

#### 4.2.2. Differences in Taxon Distribution

Beta diversity analysis characterizes the degree of difference or similarity of habitats in terms of their species composition and quantitative species representation [48]. It gives an idea of the overall diversity of community habitat conditions [49]. The beta diversity studied using the Bray-Curtis index (Figure 2) at the class level revealed that bacterial leaf dust communities not only differ from road dust communities, which was consistent with the Rome data, but they also form separate clusters according to geographic factors (bacterial leaf dust communities of Moscow clustered separately from those of Murmansk). No similar effect was observed for fungal communities. However, our study of beta diversity at a deeper taxonomic level (namely, at the species level) showed that the diversity of fungal communities was also impacted by the biotope type. We found no significant differences (*p*-value > 0.56) in beta diversity of microbial communities with respect to urban zoning in either Murmansk or Moscow (data not shown). We may conclude that the bacterial communities were the most sensitive to the anthropogenic load, biotope, and geographical factor. For example, the presence of the “polar day” in the Murmansk subarctic zone certainly influences the plants’ physiology and hence the phylloplane environment. 

We also compared the similarities of microbial species between the biotopes of the cities and found a change in the relative abundance of some bacteria in the direction of decreasing anthropogenic load from the traffic zone to the recreational zone (Figures S3 and S4). There are many degraders among *Actinobacteria*, including bacteria resistant to various pollutants [50]. Therefore, we observed an increase in the relative abundance of this class in the direction of an increasing anthropogenic gradient. As noted, we observed a high content of *Gammaproteobacteria* in the dust from leaves in the residential zone, and primarily at the expense of *Enterobacteria*. Such a phenomenon is not surprising because the members of this genus are directly related to human and animal activity [51].

The relative abundance of the species *Noviherbaspirillum suwonense* detected in all samples also increased in the direction of anthropogenic load growth in road dust in Moscow (5 times) and Murmansk (30 times). This microorganism was first isolated from air dust samples in Suwon [52]. It is known that Suwon is one of the largest centers in Korea with developed metallurgical and chemical industries. The strains of this species are likely resistant to the action of pollutants. We observed a positive correlation with W for members of the class *Betaproteobacteria*, which includes the *Noviherbaspirillum suwonense* species. 

We observed a similar pattern for the other two species belonging to the genus *Sphingomonas*. The species *Sphingomonas echinoides* increased in leaf dust in Moscow (13 times) and Murmansk (3 times), and the species *Sphingomonas solaris* increased in biotopes of both Moscow and Murmansk with the increase of the anthropogenic load. Microorganisms of the genus *Sphingomonas* are widespread in polluted sites containing toxic compounds such as polychlorinated biphenyls, creosote, pentachlorophenol, and herbicides. For example, the *S. paucimobilis* strain degrading hexachlorocyclohexane maintained a higher population density in the presence of the contaminant [53]. Similar studies demonstrated that *Sphingomonas* [54,55,56,57] can use pollutants as a source of growth and energy. The present study also revealed a correlation between both the representatives of the class *Alphaproteobacteria*, which includes the above two *Sphingomonas* species, and the species themselves with the concentration of phenanthrene, naphthalene, and some metals. 

We found no fungal communities’ species that were common to all samples and, at the same time, sensitive to anthropogenic pressure load in the functional zones of cities. Unlike fungal communities, bacterial communities are sensitive to anthropogenic load, biotope type, and geographical component. This can help to detect strains for use as bioindicators. Our results suggest focusing further attention on members of the genera *Noviherbaspirillum* and *Sphingomonas*. The indices of Chao1 richness and the Shannon diversity of fungal communities had the same dependence on metals (As, Si, Sb, K, Zn, Na, and Be) and PAHs (pyrene, phenanthrene). Whereas the index of Chao1 richness of bacterial communities depended on metals only (Nd, Pr, La, Ce, Zr, Co, Mg, Mg, Fe, and Cu), the index of Shannon diversity depended on both PAHs (anthracene, naphthalene, and phenanthrene) and metals (Pd, Hf, Ag, Ir, Ce, Ca, and U) (data not shown). Thus, anthropogenic factors, biotope type, and the functional zoning of cities affect the Chao1 richness of bacterial communities, in contrast to fungal communities. 

In the fungal communities, the number of classes positively correlated with PAH was higher than in the bacterial communities even though PAH concentrations were significantly lower than metals. In addition, we found no general pattern of the impact of either metals or PAHs on the classes of the bacterial communities of the two cities. It seems that environmental factors that influence the fungal microbiome composition are different from those that impact bacterial microbiomes [58]. For example, for contaminated soils and sediments, studies have shown that bacterial and fungal communities may exhibit differences in their response to heavy metals [59,60,61,62,63,64]. This is partly based on common differences in the biochemical pathways activated by bacteria and fungi in response to heavy metals and PAHs [65,66,67]. Undoubtedly, the pressure of external anthropogenic factors (e.g., metals, PAHs) leads to changes in the microbial community structure and the development of pollution-resistant species. Indeed, characteristic bacterial and fungal species that are “unique” to each zone were identified only in the traffic and residential zones. Thus, our study demonstrated a sensitivity of certain members of the microbial community to the content of metals/PAHs in the environment.

## 5. Conclusions

In the present work, we showed that the leaf dust had a lower alpha diversity of microbial communities than the road dust. The geographical region of the two cities did not clearly affect the fungal communities. The structure of fungal communities was more impacted by PAHs, whereas the bacterial community was more impacted by metals. The difference between the functional zones in terms of chemical composition of pollutants was confirmed, but it did not have a significant effect on the communities as a whole, affecting only certain taxa. Based on the results of this work, we suggest that bacteria of the genus *Sphingomonas* could be considered in future studies as potential bioindicators to estimate the ecological state of the urban systems. In particular, *Sphingomonas echinoides* can be considered as a potential bioindicator of PAH pollution and *Sphingomonas solaris* as a bioindicator of metals, including heavy metals (Cu, Co, Zn, Fe, Pd, Al, Mg, and W).

## Data Availability

Data set available on the NCBI SRA Portal, the accession numbers are PRJNA928599 (bacterial communities) and PRJNA928601 (fungal communities). The name of the project is “Road and leaf dust”.

## Supplementary Materials

The following supporting information can be downloaded at https://www.mdpi.com/article/10.3390/microorganisms11020526/s1. Table S1: Frequency distribution of bacterial classes/orders detected in biotopes of functional areas of Moscow and Murmansk. Table S2: Frequency distribution of fungal classes/orders detected in biotopes of functional areas of Moscow and Murmansk. Figure S1: The relative abundance (%) of the most pronounced different classes and orders of bacteria (a) and fungi (b) recovered in biotopes of functional areas of Moscow and Murmansk. The classes/orders with relative abundance ≥ 1% of the reads per given sample were included. Figure S2: Nonmetric multidimensional scaling diagram showing bacterial (A) and fungal (B) composition differences obtained among the 12 sampling sites. Figure S3: Venn diagrams illustrating the number of unique and shared bacterial OTUs among two biotopes of Moscow (A) and Murmansk (B) detected in three urban sites of cities. (C): recreational, residential and traffic zones. Figure S4: Venn diagrams illustrating the number of unique and shared fungal OTUs among two biotopes of Moscow (A) and Murmansk (B) detected in three urban sites of cities. (C): recreational, residential, and traffic zones. Figure S5: Content of the metal in biotopes of functional zones of Murmansk and Moscow. M TP, road dust from Moscow traffic zone; M RP, road dust from Moscow residential zone; M GP, road dust from Moscow recreational zone; U TP, road dust from Murmansk traffic zone; U RP, road dust from Murmansk residential zone; U GP, road dust from Murmansk recreational zone; M TL, leaf dust from Moscow traffic zone; M RL, leaf dust from Moscow residential zone; M GL, leaf dust from Moscow recreational zone; U TL, leaf dust from Murmansk traffic zone; U RL, leaf dust from Murmansk residential zone; U GL, leaf dust from Murmansk recreational zone. The red box shows the increase, and the blue box shows the decrease. Figure S6: Content of the PAHs in biotopes of functional zones of Murmansk and Moscow. M TP, road dust from Moscow traffic zone; M RP, road dust from Moscow residential zone; M GP, road dust from Moscow recreational zone; U TP, road dust from Murmansk traffic zone; U RP, road dust from Murmansk residential zone; U GP, road dust from Murmansk recreational zone; M TL, leaf dust from Moscow traffic zone; M RL, leaf dust from Moscow residential zone; M GL, leaf dust from Moscow recreational zone; U TL, leaf dust from Murmansk traffic zone; U RL, leaf dust from Murmansk residential zone; U GL, leaf dust from Murmansk recreational zone. The red box shows the increase, and the blue box shows the decrease. Figure S7: Principal component analysis (PCA) of PAHs (A) and metals (B) composition in biotopes of functional zones of Murmansk and Moscow. M–Moscow, U–Murmansk, MP–road dust of Moscow, ML–leaf dust of Moscow, UP–road dust of Murmansk, UL–leaf dust of Murmansk. Figure S8: Heat maps of bacterial (A) and fungal (B) species in microbiome of biotopes of functional zones of Murmansk and Moscow. M TP, road dust from Moscow traffic zone; M RP, road dust from Moscow residential zone; M GP, road dust from Moscow recreational zone; U TP, road dust from Murmansk traffic zone; U RP, road dust from Murmansk residential zone; U GP, road dust from Murmansk recreational zone; M TL, leaf dust from Moscow traffic zone; M RL, leaf dust from Moscow residential zone; M GL, leaf dust from Moscow recreational zone; U TL, leaf dust from Murmansk traffic zone; U RL, leaf dust from Murmansk residential zone; U GL, leaf dust from Murmansk recreational zone. The color code from pink (MIN) to red (MAX) depicts the relative amount of the respective groups. Figure S9: Heat maps of the PAHs (A) and metals (B) in biotopes of functional zones of Murmansk and Moscow. M TP, road dust from Moscow traffic zone; M RP, road dust from Moscow residential zone; M GP, road dust from Moscow recreational zone; U TP, road dust from Murmansk traffic zone; U RP, road dust from Murmansk residential zone; U GP, road dust from Murmansk recreational zone; M TL, leaf dust from Moscow traffic zone; M RL, leaf dust from Moscow residential zone; M GL, leaf dust from Moscow recreational zone; U TL, leaf dust from Murmansk traffic zone; U RL, leaf dust from Murmansk residential zone; U GL, leaf dust from Murmansk recreational zone. The color code from pink (MIN) to red (MAX) depicts the relative amount of respective groups.
