# Supplementary material for: Diversity of Microbial Communities, PAHs, and Metals in Road and Leaf Dust of Functional Zones of Moscow and Murmansk
Source: Microorganisms. 2023 Feb 18;11(2):526. doi: 10.3390/microorganisms11020526 (PMC9965023; doi:10.3390/microorganisms11020526)
Supplement: Supplementary file 1 [file microorganisms-11-00526-s001.zip › microorganisms-2164808-supplementary.pdf]

Supplementary Materials

Diversity of Microbial Communities, PAHs, and Metals in Road and Leaf Dust of Functional Zones of Moscow and Murmansk

Anna A. Vetrova [1,*], Olesya I. Sazonova [1,*], Anastasia A. Ivanova [1], Rostislav A. Streletskii [2], Dmitriy A. Sarzhanov [3], Maria V. Korneykova [3,4], Andrey I. Novikov [5], Viacheslav I. Vasenev [6], Kristina V. Ivashchenko [1,3], Marina V. Slukovskaya [3,5,7] and Olga Gavrichkova [8]

[1] Federal Research Center "Pushchino Scientific Center for Biological Research of the Russian Academy of Sciences", 142290 Pushchino, Russia
[2] Faculty of Soil Science, Laboratory of Ecological Soil Science, Lomonosov Moscow State University, 119991 Moscow, Russia
[3] Agrarian and Technological Institute, Peoples' Friendship University of Russia (RUDN University), 117198 Moscow, Russia
[4] Institute of North Industrial Ecology Problems Subdivision of the Federal Research Center "Kola Science Centre of Russian Academy of Science", 184209 Apatity, Russia
[5] I.V. Tananaev Institute of Chemistry and Technology of Rare Elements and Mineral Raw Materials, Kola Science Centre, Russian Academy of Sciences, 184209 Apatity, Russia
[6] Soil Geography and Landscape Group, Wageningen University, 6707 Wageningen, The Netherlands
[7] Laboratory of Nature-Inspired Technologies and Environmental Safety of the Arctic Region, Kola Science Centre, Russian Academy of Sciences, 184209 Apatity, Russia
[8] Research Institute on Terrestrial Ecosystems, National Research Council, 05010 Porano, Italy
* Correspondence: phdvetrova@gmail.com (A.A.V.); sazonova_oi@rambler.ru (O.I.S.)

Table S1. Frequency distribution of bacterial classes/orders detected in biotopes of functional zones of Moscow and Murmansk.

Phylum	Class / Order	Moscow						Murmansk					
		Road Dust			Leaf Dust			Road Dust			Leaf Dust		
Abditibacteriota		Recreational	Residential	Traffic	Recreational	Resuidential	Traffic	Recreational	Residential	Traffic	Recreational	Residential	Traffic
	**Abditibacteria**	**0,166**	**0,566**	**0,335**	**0.000**	**2,627**	**0,822**	**0.000**	**0.000**	**0.000**	**0,183**	**0.000**	**0.000**
	Abditibacteriales	2,065	0,623	0,486	0,017	0,023	0,160	0,018	0,142	0,307	0,002	0,003	0,009
Acidobacteria	**Acidobacteriia**	**0,130**	**0,000**	**0,153**	**2,395**	**0,110**	**0,124**	**0,122**	**0,204**	**0,081**	**0,000**	**0,758**	**0,056**
	Bryobacterales	0,002	0,009	0,000	0,000	0,001	0,000	0,012	0,001	0,001	0,000	0,001	0,001
	Acidobacteriales	0,105	0,109	0,027	0,748	0,054	0,017	2,313	0,123	0,140	0,193	0,077	0,118
	**Blastocatellia**	**1,986**	**1,817**	**0,288**	**0,021**	**0,045**	**0,053**	**0,063**	**3,728**	**1,915**	**0,009**	**0,150**	**0,166**
	Blastocatellales	1,868	1,750	0,275	0,021	0,044	0,051	0,062	3,628	1,872	0,009	0,146	0,163
	**Holophagae**	**0,000**	**0,000**	**0,000**	**0,002**	**0,000**	**0,001**	**0,000**	**0,000**	**0,000**	**0,009**	**0,000**	**0,000**
	Holophagales	0,000	0,000	0,000	0,000	0,000	0,000	0,000	0,000	0,000	0,001	0,000	0,000

	**Acidimicrobiia**	**0,330**	**0,916**	**0,214**	**0,041**	**0,059**	**0,064**	**0,019**	**2,439**	**0,732**	**0,030**	**0,297**	**0,278**
	Acidimicrobiales	0,260	0,765	0,173	0,028	0,045	0,053	0,016	2,173	0,691	0,023	0,255	0,229
	**Actinobacteria**	**12,614**	**13,250**	**19,718**	**2,761**	**3,855**	**12,608**	**13,271**	**22,920**	**20,187**	**3,161**	**6,152**	**9,851**
	Acidothermales	0,000	0,000	0,001	0,000	0,000	0,000	0,001	0,000	0,000	0,000	0,000	0,000
	Actinomycetales	0,732	1,111	0,306	0,078	0,272	0,202	1,220	0,570	0,218	0,104	0,353	0,281
	Bifidobacteriales	0,003	0,000	0,000	0,009	0,015	0,102	0,000	0,000	0,002	0,009	0,444	0,158
	Candidatus Nanopelagicales	0,000	0,000	0,000	0,005	0,003	0,010	0,000	0,000	0,000	0,003	0,004	0,002
	Corynebacteriales	0,954	1,763	2,280	0,085	0,181	0,212	2,396	2,891	5,014	0,469	0,354	1,108
	Cryptosporangiales	0,000	0,000	0,003	0,000	0,000	0,001	0,000	0,000	0,000	0,000	0,000	0,000
	Frankiales	0,095	0,065	0,060	0,007	0,022	0,092	0,161	0,099	0,149	0,011	0,028	0,037
	Geodermatophilales	1,192	0,947	2,676	0,127	0,265	0,809	0,073	0,488	1,692	0,018	0,114	0,276
	Jatrophihabitantales	0,003	0,000	0,001	0,054	0,028	0,006	1,175	0,010	0,002	0,018	0,007	0,016
	Kineosporiales	0,170	0,155	0,106	0,185	0,082	0,264	0,021	0,206	0,110	0,021	0,046	0,040
	Micrococcales	2,462	2,174	3,863	0,888	0,888	1,432	4,811	6,540	3,867	1,789	2,172	3,874
	Micromonosporales	0,006	0,006	0,011	0,009	0,015	0,008	0,018	0,032	0,003	0,018	0,015	0,032
	Motilibacterales	0,000	0,000	0,001	0,000	0,002	0,003	0,003	0,008	0,000	0,000	0,000	0,005
	Nakamurellales	0,220	0,353	0,303	0,159	0,193	4,586	2,439	1,132	0,499	0,082	0,426	0,523
	Propionibacteriales	3,715	3,976	3,857	0,927	1,208	3,671	0,343	7,505	4,889	0,242	1,376	1,850
Actinobacteria	Pseudonocardiales	0,084	0,040	0,027	0,045	0,043	0,049	0,018	0,058	0,006	0,010	0,031	0,027
	Sporichthyales	0,016	0,020	0,003	0,001	0,004	0,005	0,005	0,059	0,126	0,000	0,002	0,009
	Streptomycetales	0,068	0,063	0,012	0,021	0,011	0,024	0,003	0,061	0,010	0,046	0,029	0,040
	Streptosporangiales	0,000	0,002	0,002	0,003	0,008	0,004	0,008	0,001	0,004	0,007	0,001	0,015
	Unclassified	2,896	2,573	6,203	0,158	0,614	1,128	0,578	3,260	3,596	0,313	0,751	1,557
	**Coriobacteriia**	**0,000**	**0,000**	**0,000**	**0,000**	**0,000**	**0,000**	**0,738**	**0,280**	**1,288**	**0,000**	**0,000**	**0,000**
	Coriobacteriales	0,009	0,000	0,000	0,012	0,029	0,022	0,001	0,001	0,003	0,095	1,053	0,669
	Eggerthellales	0,000	0,000	0,000	0,002	0,009	0,004	0,000	0,000	0,000	0,023	0,166	0,027
	**Nitriliruptoria**	**0,001**	**0,000**	**0,000**	**0,000**	**0,000**	**0,001**	**0,000**	**0,000**	**0,000**	**0,000**	**0,000**	**0,001**
	Euzebyales	0,000	0,000	0,000	0,000	0,000	0,001	0,000	0,000	0,000	0,000	0,000	0,000
	**Rubrobacteria**	**0,063**	**0,048**	**0,028**	**0,003**	**0,007**	**0,019**	**0,009**	**0,078**	**0,005**	**0,010**	**0,029**	**0,030**
	Gaiellales	0,033	0,021	0,018	0,002	0,001	0,005	0,000	0,048	0,003	0,002	0,023	0,018
	Rubrobacterales	0,010	0,006	0,000	0,000	0,001	0,003	0,000	0,003	0,000	0,000	0,001	0,000
	**Thermoleophilia**	**0,364**	**0,159**	**0,070**	**0,023**	**0,029**	**0,044**	**0,033**	**0,301**	**0,078**	**0,029**	**0,062**	**0,131**
	Solirubrobacterales	0,290	0,126	0,044	0,015	0,022	0,033	0,031	0,237	0,065	0,026	0,050	0,105
Aquificae	**Aquificae**	**0,000**	**0,000**	**0,000**	**0,000**	**0,000**	**0,001**	**0,000**	**0,000**	**0,000**	**0,000**	**0,000**	**0,000**
	Aquificales	0,000	0,000	0,000	0,000	0,000	0,001	0,000	0,000	0,000	0,000	0,000	0,000

Armatimonadetes	**Armatimonadia**	**0,076**	**0,120**	**0,047**	**0,005**	**0,008**	**0,005**	**0,419**	**0,216**	**0,238**	**0,001**	**0,005**	**0,007**
	Armatimonadales	0,057	0,091	0,035	0,002	0,003	0,003	0,056	0,172	0,182	0,001	0,004	0,004
	Capsulimonadales	0,000	0,000	0,001	0,002	0,003	0,000	0,303	0,001	0,000	0,000	0,001	0,002
	**Fimbriimonadia**	**0,000**	**0,009**	**0,001**	**0,000**	**0,000**	**0,000**	**0,000**	**0,001**	**0,002**	**0,000**	**0,000**	**0,000**
	Fimbriimonadales	0,000	0,003	0,000	0,000	0,000	0,000	0,000	0,000	0,002	0,000	0,000	0,000
Bacteroidetes	**Bacteroidia**	**0,000**	**0,003**	**0,004**	**0,104**	**0,417**	**0,367**	**0,006**	**0,008**	**0,036**	**0,297**	**0,290**	**0,215**
	Bacteroidales	0,000	0,002	0,003	0,101	0,406	0,356	0,006	0,007	0,034	0,287	0,282	0,206
	Marinilabiliales	0,000	0,000	0,000	0,000	0,000	0,000	0,000	0,000	0,000	0,000	0,000	0,001
	**Chitinophagia**	**1,061**	**2,499**	**0,619**	**0,048**	**0,087**	**0,246**	**0,560**	**2,920**	**0,540**	**0,021**	**0,210**	**1,836**
	Chitinophagales	0,950	2,292	0,577	0,045	0,082	0,240	0,515	2,781	0,503	0,021	0,198	1,811
	**Cytophagia**	**10,578**	**7,502**	**8,960**	**3,053**	**1,355**	**7,467**	**10,671**	**3,785**	**7,758**	**0,415**	**0,334**	**0,594**
	Cytophagales	9,782	6,997	8,571	2,963	1,310	7,328	10,252	3,574	7,391	0,410	0,319	0,562
	**Flavobacteriia**	**1,489**	**1,410**	**0,432**	**0,052**	**0,171**	**0,112**	**0,008**	**0,533**	**0,178**	**0,033**	**0,181**	**0,328**
	Flavobacteriales	1,431	1,350	0,414	0,051	0,169	0,109	0,008	0,517	0,175	0,033	0,178	0,321
	**Saprospiria**	**0,000**	**0,001**	**0,000**	**0,000**	**0,000**	**0,001**	**0,000**	**0,000**	**0,000**	**0,000**	**0,000**	**0,002**
	Saprospirales	0,000	0,000	0,000	0,000	0,000	0,000	0,000	0,000	0,000	0,000	0,000	0,001
	**Sphingobacteriia**	**0,974**	**0,520**	**0,342**	**4,557**	**0,304**	**0,475**	**0,240**	**0,782**	**0,156**	**0,016**	**0,096**	**0,111**
	Sphingobacteriales	0,939	0,496	0,339	4,406	0,299	0,468	0,234	0,729	0,149	0,016	0,089	0,110
	Unclassified	0,008	0,011	0,001	0,002	0,000	0,000	0,000	0,006	0,000	0,000	0,000	0,000
Chlamydiae	**Chlamydiia**	**0,022**	**0,008**	**0,007**	**0,055**	**0,011**	**0,014**	**0,001**	**0,013**	**0,004**	**0,007**	**0,002**	**0,027**
	Chlamydiales	0,001	0,000	0,001	0,001	0,000	0,000	0,000	0,000	0,000	0,000	0,000	0,000
	Parachlamydiales	0,019	0,007	0,006	0,003	0,002	0,001	0,000	0,011	0,003	0,001	0,001	0,018
Chloroflexi	**Chlorobia**	**0,001**	**0,000**	**0,000**	**0,000**	**0,000**	**0,000**	**0,000**	**0,000**	**0,000**	**0,000**	**0,000**	**0,000**
	**Anaerolineae**	**0,002**	**0,001**	**0,001**	**0,001**	**0,000**	**0,000**	**0,000**	**0,003**	**0,000**	**0,000**	**0,000**	**0,000**
	**Caldilineae**	**0,001**	**0,010**	**0,000**	**0,003**	**0,000**	**0,001**	**0,000**	**0,009**	**0,000**	**0,000**	**0,002**	**0,004**
	Caldilineales	0,001	0,008	0,000	0,002	0,000	0,001	0,000	0,006	0,000	0,000	0,002	0,002
	Caldilineales	0,001	0,008	0,000	0,002	0,000	0,001	0,000	0,006	0,000	0,000	0,002	0,002
	**Candidatus Thermofonsia**	**0,001**	**0,002**	**0,000**	**0,000**	**0,000**	**0,000**	**0,000**	**0,008**	**0,000**	**0,000**	**0,004**	**0,001**
	**Chloroflexia**	**0,029**	**0,091**	**0,009**	**0,003**	**0,002**	**0,001**	**0,002**	**0,017**	**0,001**	**0,000**	**0,007**	**0,001**
	Chloroflexales	0,001	0,002	0,000	0,000	0,000	0,000	0,000	0,006	0,000	0,000	0,000	0,001
	Herpetosiphonales	0,001	0,007	0,000	0,001	0,000	0,000	0,002	0,004	0,001	0,000	0,002	0,000
	Kallotenuales	0,001	0,003	0,000	0,000	0,000	0,000	0,000	0,000	0,000	0,000	0,000	0,000
	**Ktedonobacteria**	**0,000**	**0,000**	**0,000**	**0,000**	**0,002**	**0,000**	**0,008**	**0,000**	**0,000**	**0,001**	**0,000**	**0,000**
	Ktedonobacterales	0,000	0,000	0,000	0,000	0,000	0,000	0,002	0,000	0,000	0,000	0,000	0,000

	**Tepidiformia**	**0,000**	**0,000**	**0,000**	**0,000**	**0,000**	**0,003**	**0,000**	**0,001**	**0,000**	**0,000**	**0,000**	**0,000**
	Tepidiformales	0,000	0,000	0,000	0,000	0,000	0,003	0,000	0,000	0,000	0,000	0,000	0,000
	**Thermomicrobia**	**0,009**	**0,008**	**0,001**	**0,002**	**0,000**	**0,003**	**0,000**	**0,010**	**0,001**	**0,000**	**0,000**	**0,001**
	Sphaerobacterales	0,000	0,000	0,001	0,001	0,000	0,001	0,000	0,002	0,000	0,000	0,000	0,000
Deinococcus-Thermus	**Deinococci**	**3,198**	**2,145**	**1,423**	**0,031**	**0,138**	**0,218**	**0,688**	**0,841**	**1,973**	**0,030**	**0,097**	**0,234**
	Deinococcales	2,271	1,944	1,326	0,027	0,124	0,210	0,665	0,770	1,902	0,026	0,093	0,208
	Thermales	0,000	0,000	0,000	0,000	0,000	0,000	0,000	0,000	0,000	0,000	0,000	0,001
	Trueperales	0,775	0,116	0,049	0,004	0,013	0,004	0,000	0,048	0,011	0,005	0,002	0,019
Firmicutes	**Bacilli**	**0,438**	**0,857**	**1,041**	**0,656**	**0,784**	**2,483**	**0,162**	**1,530**	**0,274**	**5,696**	**3,094**	**3,213**
	Bacillales	0,365	0,745	0,921	0,500	0,389	2,049	0,135	1,366	0,150	0,754	0,691	1,136
	Lactobacillales	0,003	0,002	0,005	0,135	0,368	0,358	0,009	0,015	0,088	4,834	2,317	1,969
	**Clostridia**	**0,057**	**0,134**	**0,031**	**0,209**	**3,798**	**0,393**	**0,143**	**0,481**	**0,173**	**2,078**	**9,247**	**2,998**
	Clostridiales	0,053	0,102	0,027	0,196	3,364	0,372	0,139	0,465	0,169	1,836	9,117	2,919
	Halanaerobiales	0,000	0,000	0,001	0,000	0,000	0,000	0,000	0,000	0,000	0,000	0,000	0,001
	Thermoanaerobacterales	0,000	0,000	0,000	0,000	0,000	0,000	0,001	0,000	0,000	0,000	0,000	0,005
	Thermosediminibacterales	0,000	0,000	0,000	0,000	0,000	0,000	0,000	0,000	0,000	0,000	0,000	0,001
	**Erysipelotrichia**	**0,005**	**0,000**	**0,000**	**0,011**	**0,067**	**0,003**	**0,005**	**0,003**	**0,026**	**0,053**	**0,479**	**0,460**
	Erysipelotrichales	0,005	0,000	0,000	0,011	0,067	0,003	0,005	0,003	0,026	0,046	0,451	0,447
	**Negativicutes**	**0,000**	**0,001**	**0,004**	**0,042**	**0,111**	**0,129**	**0,004**	**0,006**	**0,011**	**0,135**	**0,093**	**0,066**
	Acidaminococcales	0,000	0,000	0,000	0,000	0,004	0,004	0,001	0,000	0,000	0,000	0,001	0,000
	Selenomonadales	0,000	0,000	0,001	0,002	0,014	0,006	0,000	0,001	0,002	0,008	0,000	0,012
	Veillonellales	0,000	0,000	0,002	0,038	0,082	0,113	0,000	0,000	0,009	0,115	0,086	0,050
	**Tissierellia**	**0,000**	**0,001**	**0,000**	**0,009**	**0,012**	**0,009**	**0,007**	**0,011**	**0,001**	**0,011**	**0,277**	**0,030**
	Tissierellales	0,000	0,001	0,000	0,006	0,012	0,009	0,007	0,011	0,001	0,010	0,267	0,030
Fusobacteria	**Fusobacteriia**	**0,000**	**0,001**	**0,000**	**0,020**	**0,057**	**0,057**	**0,001**	**0,000**	**0,009**	**0,037**	**0,043**	**0,040**
	Fusobacteriales	0,000	0,001	0,000	0,020	0,056	0,052	0,001	0,000	0,009	0,036	0,041	0,040
Gemmatimonadetes	**Gemmatimonadetes**	**0,256**	**0,218**	**0,190**	**0,007**	**0,010**	**0,014**	**0,224**	**0,320**	**0,063**	**0,007**	**0,032**	**0,063**
	Gemmatimonadales	0,198	0,194	0,145	0,007	0,007	0,013	0,091	0,298	0,052	0,007	0,025	0,055
	**Longimicrobia**	**0,029**	**0,399**	**0,071**	**0,001**	**0,003**	**0,005**	**0,000**	**0,002**	**0,001**	**0,000**	**0,003**	**0,000**
	Longimicrobiales	0,019	0,345	0,046	0,001	0,001	0,003	0,000	0,001	0,001	0,000	0,003	0,000
Ignavibacteria	**Ignavibacteria**	**0,003**	**0,002**	**0,000**	**0,000**	**0,000**	**0,000**	**0,000**	**0,000**	**0,000**	**0,000**	**0,000**	**0,000**
Nitrospirae	**Nitrospira**	**0,008**	**0,001**	**0,001**	**0,000**	**0,000**	**0,002**	**0,000**	**0,009**	**0,000**	**0,000**	**0,000**	**0,004**
Planctomycetes	Nitrospirales	0,008	0,001	0,000	0,000	0,000	0,002	0,000	0,009	0,000	0,000	0,000	0,004

	**Phycisphaerae**	**0,372**	**0,363**	**0,423**	**0,004**	**0,004**	**0,004**	**0,044**	**0,335**	**0,271**	**0,001**	**0,005**	**0,005**
	Phycisphaerales	0,000	0,001	0,000	0,000	0,000	0,000	0,000	0,000	0,000	0,000	0,000	0,000
	Tepidisphaerales	0,241	0,216	0,255	0,003	0,002	0,003	0,032	0,117	0,084	0,000	0,002	0,004
	**Planctomycetia**	**0,834**	**0,861**	**0,685**	**0,033**	**0,068**	**0,049**	**1,211**	**1,570**	**0,676**	**0,068**	**0,180**	**0,441**
	Gemmatales	0,127	0,126	0,053	0,003	0,009	0,004	0,022	0,196	0,058	0,007	0,031	0,039
	Isosphaerales	0,124	0,104	0,213	0,006	0,014	0,010	0,460	0,420	0,292	0,025	0,050	0,248
	Pirellulales	0,005	0,005	0,004	0,000	0,003	0,004	0,001	0,014	0,001	0,002	0,008	0,013
	Planctomycetales	0,185	0,123	0,106	0,011	0,017	0,012	0,541	0,316	0,124	0,024	0,041	0,071
Proteobacteria	**Alphaproteobacteria**	**18,862**	**17,644**	**22,523**	**16,813**	**5,526**	**10,322**	**26,261**	**14,344**	**21,103**	**3,859**	**4,391**	**10,761**
	Caulobacterales	0,672	0,426	0,490	0,019	0,052	0,070	0,245	0,421	0,209	0,077	0,089	0,539
	Holosporales	0,005	0,000	0,003	0,003	0,004	0,002	0,001	0,000	0,003	0,002	0,001	0,000
	Hyphomonadales	0,002	0,003	0,000	0,000	0,001	0,001	0,000	0,004	0,000	0,000	0,001	0,004
	Micropepsales	0,008	0,001	0,001	0,000	0,000	0,000	0,001	0,000	0,000	0,000	0,000	0,002
	Rhizobiales	1,750	1,229	2,249	1,355	0,631	1,491	5,600	2,025	3,867	0,920	0,872	5,863
	Rhodobacterales	4,948	5,984	3,454	0,217	0,959	0,655	0,021	1,273	0,240	0,073	0,386	0,517
	Rhodospirillales	4,592	3,651	7,681	10,982	2,134	3,059	14,472	3,531	7,026	2,208	1,691	1,423
	Rickettsiales	0,000	0,000	0,005	0,016	0,024	0,035	0,005	0,002	0,006	0,037	0,004	0,040
	Sphingomonadales	4,883	4,767	6,903	3,656	1,490	4,749	4,419	5,843	8,525	0,307	1,166	1,779
	**Betaproteobacteria**	**3,811**	**6,912**	**4,923**	**1,188**	**1,213**	**2,511**	**2,518**	**7,162**	**8,209**	**0,478**	**2,003**	**5,894**
	Burkholderiales	2,096	4,899	4,434	1,075	0,968	2,277	1,771	5,882	6,436	0,368	1,746	5,575
	Neisseriales	0,003	0,006	0,001	0,028	0,048	0,102	0,002	0,003	0,002	0,048	0,021	0,081
	Nitrosomonadales	0,017	0,018	0,001	0,001	0,005	0,007	0,006	0,009	0,003	0,001	0,007	0,007
	Procabacteriales	0,000	0,000	0,000	0,000	0,000	0,000	0,000	0,002	0,001	0,000	0,000	0,000
	Rhodocyclales	0,000	0,002	0,002	0,000	0,000	0,001	0,000	0,000	0,001	0,001	0,001	0,001
	**Deltaproteobacteria**	**0,290**	**0,764**	**0,147**	**0,291**	**0,123**	**0,088**	**0,307**	**0,367**	**0,280**	**1,151**	**0,461**	**0,536**
	Desulfobacterales	0,000	0,000	0,000	0,000	0,000	0,000	0,000	0,000	0,001	0,000	0,001	0,000
	Desulfovibrionales	0,000	0,000	0,000	0,000	0,001	0,000	0,000	0,000	0,000	0,000	0,000	0,000
	Desulfuromonadales	0,003	0,007	0,003	0,011	0,002	0,003	0,010	0,012	0,008	0,002	0,003	0,053
	Myxococcales	0,105	0,426	0,047	0,010	0,010	0,040	0,026	0,130	0,007	0,017	0,040	0,087
	**Epsilonproteobacteria**	**0,000**	**0,000**	**0,000**	**0,000**	**0,000**	**0,007**	**0,000**	**0,001**	**0,001**	**0,000**	**0,002**	**0,005**
	Campylobacterales	0,000	0,000	0,000	0,000	0,000	0,007	0,000	0,001	0,001	0,000	0,002	0,005
	**Gammaproteobacteria**	**0,759**	**1,157**	**2,499**	**18,602**	**27,560**	**20,197**	**0,170**	**1,563**	**1,598**	**12,805**	**18,438**	**4,573**
	Acidiferrobacterales	0,000	0,000	0,000	0,000	0,000	0,001	0,000	0,000	0,000	0,000	0,000	0,000
	Aeromonadales	0,001	0,005	0,000	0,000	0,001	0,000	0,000	0,000	0,002	0,003	0,023	0,002
	Alteromonadales	0,000	0,000	0,001	0,000	0,008	0,000	0,000	0,001	0,000	0,000	0,000	0,006

	Cardiobacteriales	0,000	0,000	0,000	0,000	0,001	0,000	0,000	0,000	0,000	0,000	0,000	0,000
	Cellvibrionales	0,023	0,042	0,005	0,000	0,001	0,002	0,001	0,004	0,009	0,000	0,003	0,005
	Chromatiales	0,005	0,047	0,015	0,005	0,003	0,007	0,000	0,001	0,003	0,002	0,009	0,041
	Enterobacterales	0,003	0,009	0,007	15,601	25,240	9,782	0,009	0,003	0,011	8,728	17,420	2,027
	Immundisolibacterales	0,002	0,012	0,000	0,000	0,001	0,000	0,000	0,004	0,000	0,000	0,000	0,004
	Legionellales	0,003	0,020	0,009	0,003	0,002	0,004	0,037	0,013	0,018	0,017	0,013	0,020
	Methylococcales	0,000	0,000	0,000	0,000	0,002	0,000	0,000	0,001	0,000	0,001	0,003	0,001
	Nevskiales	0,000	0,000	0,000	0,000	0,000	0,003	0,003	0,002	0,001	0,000	0,000	1,406
	Oceanospirillales	0,001	0,000	0,001	0,003	0,000	0,002	0,000	0,004	0,000	0,000	0,000	0,021
	Pasteurellales	0,001	0,000	0,001	0,000	0,018	0,008	0,000	0,000	0,003	0,005	0,008	0,036
	Pseudomonadales	0,073	0,435	2,170	0,315	1,052	1,352	0,015	0,107	1,057	2,105	0,188	0,557
	Thiotrichales	0,000	0,000	0,000	0,001	0,000	0,000	0,000	0,000	0,001	0,000	0,000	0,002
	Vibrionales	0,000	0,000	0,000	0,000	0,000	0,000	0,000	0,000	0,000	0,000	0,000	0,021
	Xanthomonadales	0,602	0,513	0,204	0,013	0,043	0,045	0,069	1,344	0,427	0,020	0,159	0,298
	**Hydrogenophilalia**	**0,000**	**0,000**	**0,000**	**0,000**	**0,001**	**0,003**	**0,000**	**0,000**	**0,000**	**0,000**	**0,000**	**0,000**
	Hydrogenophilales	0,000	0,000	0,000	0,000	0,001	0,003	0,000	0,000	0,000	0,000	0,000	0,000
	**Oligoflexia**	**0,293**	**1,598**	**1,007**	**0,038**	**0,053**	**0,041**	**0,046**	**1,591**	**0,426**	**0,032**	**0,124**	**0,247**
	Bacteriovoracales	0,084	0,179	0,117	0,001	0,009	0,008	0,001	0,256	0,071	0,005	0,010	0,036
	Bdellovibrionales	0,074	0,927	0,689	0,024	0,024	0,018	0,014	0,769	0,159	0,017	0,062	0,133
	Oligoflexales	0,004	0,063	0,002	0,003	0,003	0,003	0,001	0,057	0,002	0,003	0,005	0,005
	Silvanigrellales	0,020	0,068	0,001	0,000	0,000	0,000	0,001	0,001	0,003	0,001	0,000	0,000
Rhodothermaeota	**Rhodothermia**	**0,496**	**0,127**	**0,116**	**0,002**	**0,009**	**0,003**	**0,001**	**0,311**	**0,036**	**0,000**	**0,017**	**0,014**
	Rhodothermales	0,461	0,118	0,105	0,001	0,009	0,003	0,001	0,295	0,035	0,000	0,016	0,014
	**Spirochaetia**	**0,000**	**0,000**	**0,001**	**0,000**	**0,000**	**0,001**	**0,001**	**0,000**	**0,000**	**0,003**	**0,001**	**0,000**
	Leptospirales	0,000	0,000	0,001	0,000	0,000	0,000	0,000	0,000	0,000	0,000	0,000	0,000
	Spirochaetales	0,000	0,000	0,000	0,000	0,000	0,001	0,001	0,000	0,000	0,003	0,001	0,000
Spirochaetes	**Mollicutes**	**0,096**	**0,303**	**0,382**	**2,371**	**0,310**	**3,024**	**0,156**	**0,444**	**0,419**	**4,050**	**4,335**	**4,796**
	Acholeplasmatales	0,092	0,266	0,358	2,313	0,260	2,980	0,036	0,417	0,394	3,939	4,251	4,724
	Entomoplasmatales	0,000	0,000	0,000	0,000	0,001	0,000	0,000	0,000	0,000	0,000	0,000	0,000
	Mycoplasmatales	0,000	0,000	0,000	0,000	0,000	0,001	0,000	0,000	0,000	0,005	0,001	0,005
	**Opitutae**	**0,030**	**0,051**	**0,006**	**0,001**	**0,007**	**0,003**	**0,000**	**0,016**	**0,001**	**0,002**	**0,010**	**0,004**
	**Spartobacteria**	**0,604**	**0,971**	**0,195**	**0,010**	**0,032**	**0,016**	**0,129**	**0,301**	**0,055**	**0,011**	**0,037**	**0,044**
Verrucomicrobia	**Terrimicrobia**	**0,000**	**0,000**	**0,000**	**0,000**	**0,000**	**0,000**	**0,000**	**0,000**	**0,000**	**0,001**	**0,000**	**0,001**
	**Verrucomicrobiae**	**0,208**	**0,730**	**0,143**	**0,012**	**0,014**	**0,032**	**0,006**	**0,338**	**0,081**	**0,009**	**0,062**	**0,076**
	**Verrucomicrobiae**	**0,338**	**0,143**	**0,081**	**0,000**	**0,208**	**0,730**	**0,076**	**0,000**	**0,062**	**0,000**	**0,000**	**0,000**

	Verrucomicrobiales	0,187	0,682	0,131	0,011	0,013	0,031	0,005	0,308	0,074	0,008	0,052	0,069

Table S2. Frequency distribution of fungal classes/orders detected in biotopes of functional zones of Moscow and Murmansk.

Phylum	Class / Order	Moscow						Murmansk					
		Road Dust			Leaf Dust			Road Dust			Leaf Dust		
		Recreational	Residential	Traffic	Recreational	Residential	Traffic	Recreational	Residential	Traffic	Recreational	Residential	Traffic
	**Archaeorhizomycetes**	**0,000**	**0,000**	**0,000**	**0,000**	**0,000**	**0,000**	**0,000**	**0,004**	**0,000**	**0,000**	**0,000**	**0,000**
	Archaeorhizomycetales	0,000	0,000	0,000	0,000	0,000	0,000	0,000	0,004	0,000	0,000	0,000	0,000
	Arthoniales	0,000	0,000	0,000	0,000	0,000	0,000	0,000	0,004	0,000	0,000	0,000	0,000
	Lichenostigmatales	0,000	0,000	0,023	0,000	0,030	0,048	0,038	0,045	0,043	0,081	0,045	0,004
	**Arthoniomycetes**	**0,000**	**0,000**	**0,029**	**0,010**	**0,043**	**0,053**	**0,131**	**0,134**	**0,082**	**0,306**	**0,048**	**0,005**
	**Candelariomycetes**	**0,013**	**0,014**	**0,000**	**0,000**	**0,000**	**0,000**	**0,005**	**0,007**	**0,016**	**0,000**	**0,000**	**0,002**
	Candelariales	0,013	0,014	0,000	0,000	0,000	0,000	0,005	0,007	0,016	0,000	0,000	0,002
	**Dothideomycetes**	**40,301**	**33,345**	**57,803**	**33,277**	**30,795**	**61,419**	**3,948**	**29,113**	**18,932**	**43,005**	**58,891**	**83,629**
	Abrothallales	0,000	0,000	0,000	0,000	0,000	0,000	0,009	0,000	0,035	0,000	0,000	0,000
	Acrospermales	0,027	0,000	0,003	0,000	0,000	0,005	0,000	0,000	0,000	0,000	0,000	0,000
	Botryosphaeriales	0,453	0,569	1,323	0,307	0,105	0,300	0,005	0,030	0,020	0,029	0,020	0,009
	Capnodiales	0,067	0,007	0,013	0,000	0,000	0,005	0,005	0,004	0,008	0,011	0,002	0,019
Ascomycota	Cladosporiales	2,396	1,261	0,696	0,456	1,174	0,161	0,150	3,717	1,299	0,299	0,416	0,167
	Dothideales	2,729	2,001	7,174	13,466	11,586	21,731	0,671	8,236	3,665	27,955	23,793	31,066
	Hysteriales	0,000	0,000	0,000	0,000	0,003	0,000	0,000	0,000	0,000	0,000	0,000	0,000
	Muyocopronales	0,027	0,021	0,023	0,000	0,000	0,000	0,000	0,004	0,000	0,000	0,000	0,000
	Mycosphaerellales	1,731	0,528	0,356	0,362	0,469	0,070	0,108	0,816	1,954	0,269	0,139	0,835
	Myriangiales	0,013	0,034	0,168	0,045	0,022	0,380	0,249	0,037	0,043	0,260	0,436	0,000
	Mytilinidiales	0,080	0,048	0,000	0,000	0,008	0,000	0,000	0,004	0,000	0,000	0,000	0,000
	Neophaeothecales	0,000	0,000	0,000	0,000	0,000	0,000	0,000	0,000	0,086	0,000	0,000	0,000
	Phaeothecales	0,000	0,000	0,000	0,000	0,003	0,000	0,080	0,004	0,043	0,111	0,028	0,104
	Pleosporales	18,293	15,720	23,821	2,731	3,839	2,049	0,333	5,023	3,700	0,283	0,628	0,114
	Strigulales	0,000	0,000	0,000	0,000	0,003	0,000	0,000	0,000	0,000	0,002	0,000	0,000
	Tubeufiales	0,013	0,007	0,000	0,000	0,000	0,000	0,005	0,000	0,016	0,000	0,000	0,000
	Unclassified	13,993	12,725	23,983	14,691	12,973	36,534	0,685	7,728	0,895	11,276	32,973	51,136
	Venturiales	0,479	0,425	0,243	1,219	0,611	0,185	1,648	3,510	7,169	2,512	0,456	0,179
	**Eurotiomycetes**	**15,138**	**25,416**	**6,643**	**7,613**	**3,378**	**2,323**	**52,164**	**13,108**	**24,037**	**2,854**	**2,342**	**0,541**

Chaetothyriales	8,334	23,429	5,821	5,412	2,333	2,117	48,841	11,742	22,828	1,352	1,476	0,160
Eurotiales	0,120	0,130	0,074	0,134	0,151	0,115	0,023	0,156	0,110	0,043	0,165	0,049
Mycocaliciales	0,000	0,000	0,029	0,159	0,291	0,010	0,009	0,000	0,024	0,004	0,015	0,235
Onygenales	0,013	0,007	0,000	0,000	0,000	0,000	0,000	0,030	0,000	0,004	0,000	0,000
Phaeomoniellales	0,000	0,000	0,000	0,059	0,008	0,010	0,028	0,004	0,004	0,038	0,003	0,000
Verrucariales	5,232	0,185	0,320	0,000	0,016	0,000	0,084	0,386	0,432	0,034	0,046	0,002
**Lecanoromycetes**	**9,985**	**11,129**	**3,297**	**6,974**	**1,153**	**0,216**	**11,528**	**5,387**	**29,397**	**0,256**	**0,144**	**0,021**
Acarosporales	0,040	0,000	0,042	0,015	0,013	0,014	0,000	0,015	0,055	0,002	0,009	0,000
Baeomycetales	0,000	0,000	0,000	0,000	0,000	0,000	0,014	0,000	0,000	0,000	0,000	0,000
Caliciales	0,479	0,116	0,019	0,010	0,035	0,002	0,000	0,000	0,004	0,000	0,000	0,002
Lecanorales	0,240	0,082	0,045	0,010	0,035	0,010	0,314	0,364	0,844	0,005	0,002	0,000
Lecideales	0,000	0,000	0,000	0,000	0,000	0,000	0,272	0,000	0,024	0,000	0,000	0,000
Ostropales	0,000	0,000	0,000	0,010	0,000	0,000	0,005	0,000	0,000	0,002	0,002	0,000
Peltigerales	0,013	0,000	0,000	0,000	0,000	0,002	0,000	0,000	0,000	0,000	0,000	0,000
Pertusariales	0,000	0,000	0,003	0,000	0,019	0,002	0,005	0,000	0,000	0,002	0,000	0,000
Rhizocarpales	0,000	0,000	0,000	0,000	0,000	0,000	0,005	0,000	0,004	0,000	0,000	0,000
Teloschistales	7,655	10,156	2,394	5,893	0,800	0,099	3,689	4,014	27,407	0,131	0,065	0,016
Trapeliales	0,000	0,000	0,000	0,005	0,000	0,000	0,028	0,004	0,000	0,007	0,011	0,000
Umbilicariales	0,027	0,014	0,081	0,069	0,054	0,043	0,155	0,019	0,020	0,021	0,013	0,000
**Leotiomycetes**	**5,139**	**2,864**	**1,854**	**2,156**	**4,876**	**0,788**	**2,446**	**9,156**	**4,591**	**11,891**	**1,157**	**0,165**
Chaetomellales	0,000	0,034	0,000	0,000	0,000	0,000	0,000	0,000	0,000	0,000	0,000	0,000
Erysiphales	0,080	0,178	0,152	0,421	2,152	0,247	0,005	0,063	0,141	0,000	0,082	0,005
Helotiales	4,593	1,953	1,365	1,482	1,848	0,471	1,854	5,806	2,743	8,641	0,636	0,125
Leotiales	0,000	0,007	0,000	0,000	0,005	0,000	0,066	0,007	0,024	0,013	0,003	0,000
Marthamycetales	0,000	0,000	0,003	0,010	0,003	0,000	0,000	0,000	0,000	0,000	0,000	0,000
Phacidiales	0,000	0,034	0,006	0,000	0,003	0,000	0,014	0,004	0,008	0,023	0,000	0,000
Rhytismatales	0,013	0,000	0,000	0,000	0,000	0,002	0,014	0,267	0,149	0,059	0,035	0,004
Thelebolales	0,266	0,281	0,071	0,005	0,003	0,005	0,047	1,688	0,679	0,007	0,007	0,005
**Orbiliomycetes**	**0,067**	**0,014**	**0,023**	**0,000**	**0,043**	**0,010**	**0,000**	**0,000**	**0,027**	**0,000**	**0,000**	**0,000**
Orbiliales	0,067	0,014	0,013	0,000	0,030	0,002	0,000	0,000	0,027	0,000	0,000	0,000
**Pezizomycetes**	**0,053**	**0,048**	**0,016**	**0,050**	**0,059**	**0,026**	**0,005**	**0,193**	**0,055**	**0,023**	**0,030**	**0,000**
Pezizales	0,053	0,041	0,013	0,050	0,048	0,024	0,005	0,122	0,043	0,005	0,012	0,000
**Saccharomycetes**	**0,067**	**0,062**	**0,081**	**0,020**	**0,011**	**0,017**	**0,019**	**0,271**	**0,102**	**0,082**	**0,047**	**0,629**
Saccharomycetales	0,040	0,062	0,074	0,020	0,005	0,017	0,019	0,260	0,102	0,056	0,030	0,601

	**Sordariomycetes**	**0,266**	**0,343**	**0,181**	**0,496**	**0,312**	**0,555**	**0,084**	**0,427**	**0,259**	**0,066**	**0,067**	**0,033**
	Chaetosphaeriales	0,013	0,048	0,000	0,000	0,000	0,007	0,000	0,004	0,008	0,000	0,000	0,000
	Coniochaetales	0,000	0,000	0,000	0,000	0,000	0,000	0,000	0,000	0,004	0,002	0,001	0,000
	Diaporthales	0,000	0,007	0,042	0,367	0,210	0,449	0,005	0,022	0,051	0,043	0,034	0,025
	Glomerellales	0,013	0,007	0,003	0,000	0,000	0,000	0,000	0,037	0,004	0,011	0,004	0,000
	Hypocreales	0,107	0,082	0,052	0,069	0,046	0,055	0,038	0,178	0,126	0,011	0,021	0,005
	Microascales	0,000	0,000	0,006	0,000	0,000	0,000	0,000	0,011	0,000	0,000	0,000	0,000
	Ophiostomatales	0,000	0,007	0,000	0,000	0,000	0,000	0,000	0,004	0,000	0,000	0,000	0,000
	Sordariales	0,000	0,007	0,019	0,000	0,000	0,000	0,005	0,019	0,008	0,000	0,001	0,000
	Togniniales	0,000	0,000	0,000	0,000	0,000	0,002	0,000	0,000	0,000	0,000	0,000	0,000
	Xenospadicoidales	0,000	0,000	0,000	0,000	0,000	0,000	0,000	0,000	0,004	0,000	0,000	0,000
	Xylariales	0,067	0,082	0,006	0,005	0,019	0,000	0,019	0,093	0,008	0,000	0,000	0,002
	**Taphrinomycetes**	**0,253**	**0,116**	**0,210**	**3,013**	**6,802**	**2,304**	**0,488**	**0,382**	**1,946**	**9,158**	**5,735**	**1,379**
	Taphrinales	0,240	0,116	0,204	2,964	6,683	2,302	0,488	0,382	1,942	7,805	5,707	1,378
	Unclassified	10,451	10,683	12,315	19,935	13,272	13,606	17,635	11,445	5,427	17,121	17,321	6,033
	Symbiotaphrinales	0,000	0,000	0,000	0,000	0,003	0,000	0,000	0,000	0,000	0,000	0,000	0,000
	**Agaricomycetes**	**2,077**	**0,987**	**0,201**	**0,322**	**0,253**	**0,197**	**0,169**	**2,297**	**0,608**	**0,077**	**0,129**	**0,019**
	Agaricales	0,746	0,459	0,049	0,124	0,051	0,050	0,108	1,974	0,314	0,059	0,093	0,014
	Amylocorticiales	0,000	0,007	0,000	0,000	0,000	0,000	0,000	0,026	0,000	0,000	0,000	0,000
	Atheliales	0,000	0,007	0,000	0,000	0,000	0,000	0,000	0,004	0,004	0,000	0,000	0,000
	Auriculariales	0,027	0,000	0,003	0,005	0,003	0,029	0,005	0,011	0,020	0,000	0,001	0,000
	Boletales	0,013	0,014	0,003	0,000	0,003	0,000	0,000	0,007	0,020	0,000	0,010	0,000
	Cantharellales	0,093	0,055	0,013	0,000	0,003	0,002	0,009	0,067	0,020	0,000	0,001	0,000
	Corticiales	0,040	0,021	0,003	0,015	0,046	0,010	0,000	0,000	0,047	0,002	0,003	0,000
Basidiomycota	Gloeophyllales	0,000	0,000	0,000	0,000	0,000	0,000	0,000	0,000	0,008	0,000	0,000	0,000
	Gomphales	0,000	0,000	0,000	0,000	0,000	0,000	0,000	0,007	0,000	0,000	0,000	0,000
	Hymenochaetales	0,040	0,021	0,000	0,005	0,005	0,002	0,000	0,026	0,008	0,000	0,000	0,000
	Phallales	0,067	0,000	0,000	0,000	0,000	0,000	0,000	0,004	0,000	0,000	0,000	0,000
	Polyporales	0,599	0,206	0,094	0,139	0,108	0,084	0,000	0,100	0,094	0,004	0,007	0,002
	Russulales	0,027	0,110	0,003	0,015	0,024	0,014	0,000	0,026	0,047	0,005	0,001	0,000
	Sebacinales	0,000	0,000	0,003	0,000	0,000	0,000	0,009	0,004	0,008	0,000	0,000	0,000
	Thelephorales	0,120	0,007	0,013	0,000	0,003	0,000	0,005	0,004	0,004	0,000	0,001	0,000
	Trechisporales	0,053	0,007	0,000	0,000	0,000	0,000	0,009	0,011	0,000	0,000	0,001	0,000
	**Agaricostilbomycetes**	**0,186**	**0,110**	**0,366**	**0,074**	**0,420**	**0,459**	**0,033**	**0,096**	**0,286**	**0,030**	**0,101**	**0,004**

	Agaricostilbales	0,160	0,082	0,233	0,040	0,377	0,135	0,033	0,074	0,255	0,007	0,009	0,004
	**Atractiellomycetes**	**0,000**	**0,000**	**0,000**	**0,010**	**0,000**	**0,002**	**0,000**	**0,000**	**0,000**	**0,000**	**0,004**	**0,000**
	Atractiellales	0,000	0,000	0,000	0,000	0,000	0,002	0,000	0,000	0,000	0,000	0,000	0,000
	**Cystobasidiomycetes**	**0,932**	**0,726**	**1,288**	**0,748**	**0,412**	**1,999**	**0,221**	**1,028**	**1,864**	**0,381**	**0,494**	**0,380**
	Cystobasidiales	0,240	0,171	0,579	0,030	0,013	0,058	0,066	0,482	1,734	0,029	0,009	0,026
	Erythrobasidiales	0,067	0,021	0,019	0,000	0,005	0,310	0,000	0,004	0,000	0,002	0,000	0,000
	**Dacrymycetes**	**0,013**	**0,027**	**0,000**	**0,000**	**0,000**	**0,000**	**0,000**	**0,004**	**0,000**	**0,000**	**0,001**	**0,000**
	Dacrymycetales	0,013	0,007	0,000	0,000	0,000	0,000	0,000	0,004	0,000	0,000	0,001	0,000
	**Exobasidiomycetes**	**0,226**	**0,089**	**0,094**	**2,280**	**3,176**	**1,007**	**0,042**	**0,011**	**0,027**	**0,091**	**0,060**	**0,007**
	Entylomatales	0,000	0,000	0,000	0,000	0,000	0,000	0,005	0,007	0,004	0,004	0,000	0,000
	Exobasidiales	0,013	0,007	0,000	0,005	0,003	0,000	0,033	0,004	0,024	0,082	0,049	0,005
	Golubeviales	0,000	0,000	0,000	0,000	0,000	0,000	0,000	0,000	0,000	0,002	0,000	0,000
	Microstromatales	0,200	0,082	0,094	2,250	3,149	1,007	0,000	0,000	0,000	0,000	0,010	0,002
	**Malasseziomycetes**	**0,000**	**0,000**	**0,003**	**0,000**	**0,032**	**0,007**	**0,000**	**0,004**	**0,027**	**0,000**	**0,000**	**0,002**
	Malasseziales	0,000	0,000	0,003	0,000	0,030	0,007	0,000	0,004	0,027	0,000	0,000	0,002
	**Microbotryomycetes**	**0,679**	**0,706**	**0,692**	**0,129**	**0,027**	**0,190**	**0,352**	**2,538**	**1,613**	**0,030**	**0,010**	**0,004**
	Kriegeriales	0,546	0,418	0,586	0,000	0,000	0,002	0,207	1,670	1,428	0,002	0,001	0,000
	Leucosporidiales	0,040	0,116	0,016	0,000	0,005	0,000	0,014	0,364	0,067	0,025	0,000	0,000
	Microbotryales	0,040	0,062	0,013	0,000	0,000	0,000	0,009	0,063	0,024	0,000	0,001	0,000
	Sporidiobolales	0,013	0,055	0,049	0,114	0,019	0,183	0,014	0,096	0,027	0,000	0,008	0,004
	**Pucciniomycetes**	**0,213**	**0,219**	**0,039**	**0,248**	**1,086**	**0,368**	**0,314**	**0,434**	**1,263**	**1,457**	**0,205**	**0,580**
	Pucciniales	0,213	0,219	0,039	0,248	1,080	0,365	0,310	0,434	1,263	1,454	0,198	0,578
	Septobasidiales	0,000	0,000	0,000	0,000	0,000	0,002	0,000	0,000	0,000	0,000	0,004	0,002
	**Spiculogloeomycetes**	**0,013**	**0,000**	**0,003**	**0,025**	**0,000**	**0,000**	**0,000**	**0,000**	**0,024**	**0,000**	**0,001**	**0,000**
	Cystofilobasidiales	0,120	0,247	0,841	0,020	0,048	0,824	0,047	2,604	0,742	0,258	0,072	0,079
	Filobasidiales	0,519	0,699	2,427	0,352	0,504	2,857	0,169	2,693	0,734	0,267	0,422	0,051
	Holtermanniales	0,013	0,027	0,013	0,000	0,000	0,000	0,000	0,122	0,020	0,000	0,006	0,000
	**Tremellomycetes**	**1,891**	**1,727**	**4,465**	**2,686**	**2,643**	**5,278**	**1,080**	**8,663**	**3,433**	**1,885**	**0,868**	**0,297**
	Tremellales	1,145	0,500	0,550	2,097	1,886	1,516	0,667	1,447	1,040	1,329	0,334	0,146
	Trichosporonales	0,000	0,000	0,000	0,000	0,003	0,000	0,000	0,037	0,016	0,000	0,000	0,000
	**Ustilaginomycetes**	**0,027**	**0,075**	**0,006**	**0,025**	**0,194**	**0,002**	**0,000**	**0,000**	**0,024**	**0,002**	**0,002**	**0,004**
	Urocystidales	0,027	0,055	0,000	0,005	0,137	0,000	0,000	0,000	0,024	0,000	0,002	0,004
	Ustilaginales	0,000	0,021	0,006	0,020	0,057	0,002	0,000	0,000	0,000	0,002	0,000	0,000
Blastocladiomycota	**Blastocladiomycetes**	**0,013**	**0,007**	**0,000**	**0,000**	**0,000**	**0,000**	**0,000**	**0,000**	**0,000**	**0,000**	**0,000**	**0,000**

	Blastocladiales	0,013	0,007	0,000	0,000	0,000	0,000	0,000	0,000	0,000	0,000	0,000	0,000
Chytridiomycota	**Chytridiomycetes**	**0,772**	**1,775**	**0,388**	**0,000**	**0,003**	**0,000**	**0,014**	**1,336**	**0,149**	**0,000**	**0,000**	**0,000**
	Rhizophlyctidales	0,000	0,000	0,003	0,000	0,000	0,000	0,000	0,000	0,000	0,000	0,000	0,000
	Rhizophydiales	0,000	0,041	0,003	0,000	0,000	0,000	0,000	0,004	0,000	0,000	0,000	0,000
	Spizellomycetales	0,692	1,651	0,369	0,000	0,003	0,000	0,009	1,206	0,137	0,000	0,000	0,000
	**Monoblepharidomycetes**	**0,027**	**0,000**	**0,000**	**0,000**	**0,000**	**0,000**	**0,000**	**0,000**	**0,000**	**0,000**	**0,000**	**0,000**
	Monoblepharidales	0,013	0,000	0,000	0,000	0,000	0,000	0,000	0,000	0,000	0,000	0,000	0,000
	**Neocallimastigomycetes**	**0,000**	**0,000**	**0,000**	**0,005**	**0,000**	**0,000**	**0,000**	**0,000**	**0,000**	**0,000**	**0,000**	**0,002**
	Neocallimastigales	0,000	0,000	0,000	0,005	0,000	0,000	0,000	0,000	0,000	0,000	0,000	0,002
Mucoromycota	**Glomeromycetes**	**0,107**	**0,048**	**0,026**	**0,030**	**0,000**	**0,000**	**0,056**	**0,033**	**0,000**	**0,000**	**0,000**	**0,000**
	Diversisporales	0,080	0,034	0,006	0,000	0,000	0,000	0,028	0,015	0,000	0,000	0,000	0,000
	Glomerales	0,013	0,007	0,003	0,025	0,000	0,000	0,009	0,007	0,000	0,000	0,000	0,000
	Paraglomerales	0,000	0,000	0,000	0,000	0,000	0,000	0,000	0,004	0,000	0,000	0,000	0,000
	**Mortierellomycetes**	**0,027**	**0,021**	**0,003**	**0,005**	**0,003**	**0,000**	**0,000**	**0,007**	**0,000**	**0,007**	**0,003**	**0,000**
	Mortierellales	0,027	0,021	0,003	0,005	0,003	0,000	0,000	0,004	0,000	0,007	0,002	0,000
	**Mucoromycetes**	**0,027**	**0,110**	**0,003**	**0,015**	**0,054**	**0,005**	**0,005**	**0,048**	**0,027**	**0,021**	**0,006**	**0,005**
	Mucorales	0,027	0,110	0,003	0,015	0,054	0,005	0,005	0,048	0,027	0,021	0,006	0,005

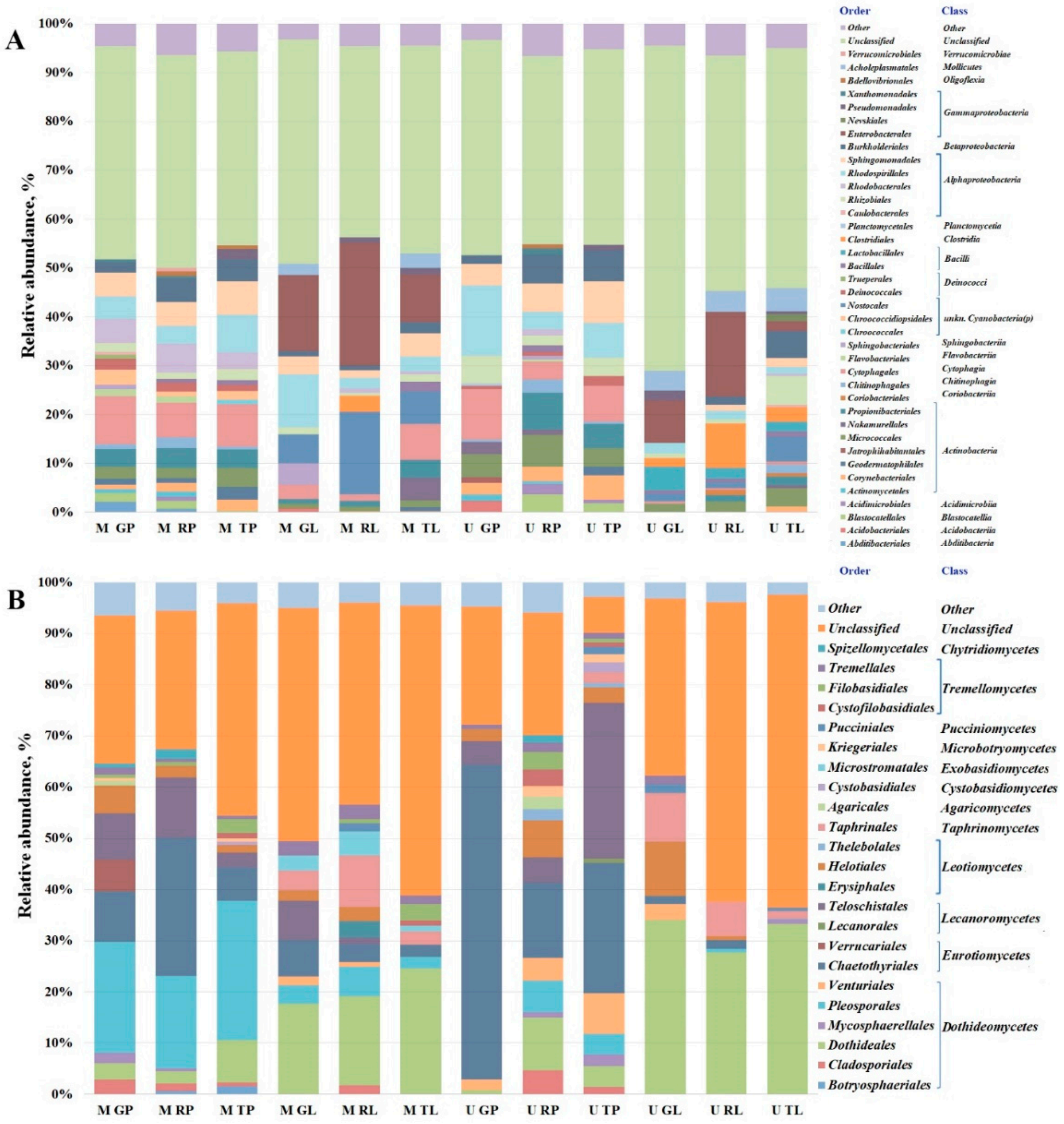

Figure S1. The relative abundance (%) of the most pronounced different classes and orders of bacteria (**a**) and fungi (**b**) recovered in biotopes of functional zones of Moscow and Murmansk. The classes/orders with relative abundance ≥ 1% of the reads per given sample were included.

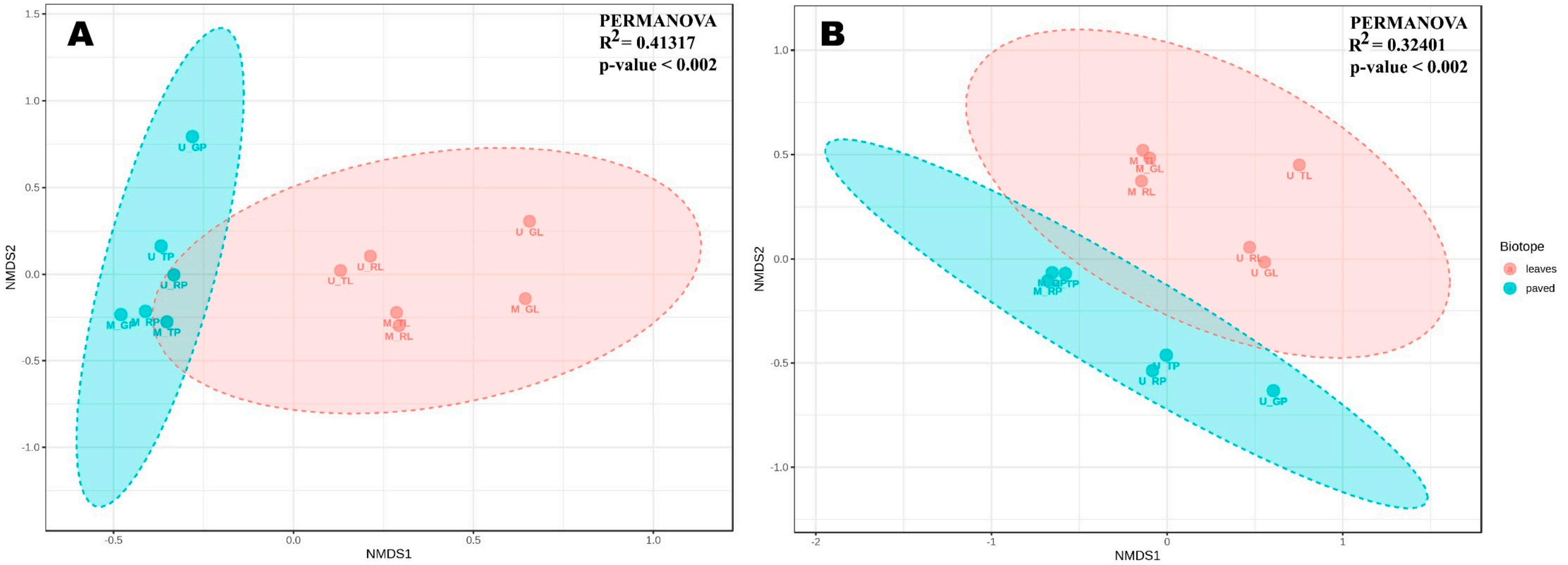

Figure S2. Non-metric multidimensional scaling diagram showing bacterial (A) and fungal (B) composition differences obtained among the 12 sampling sites

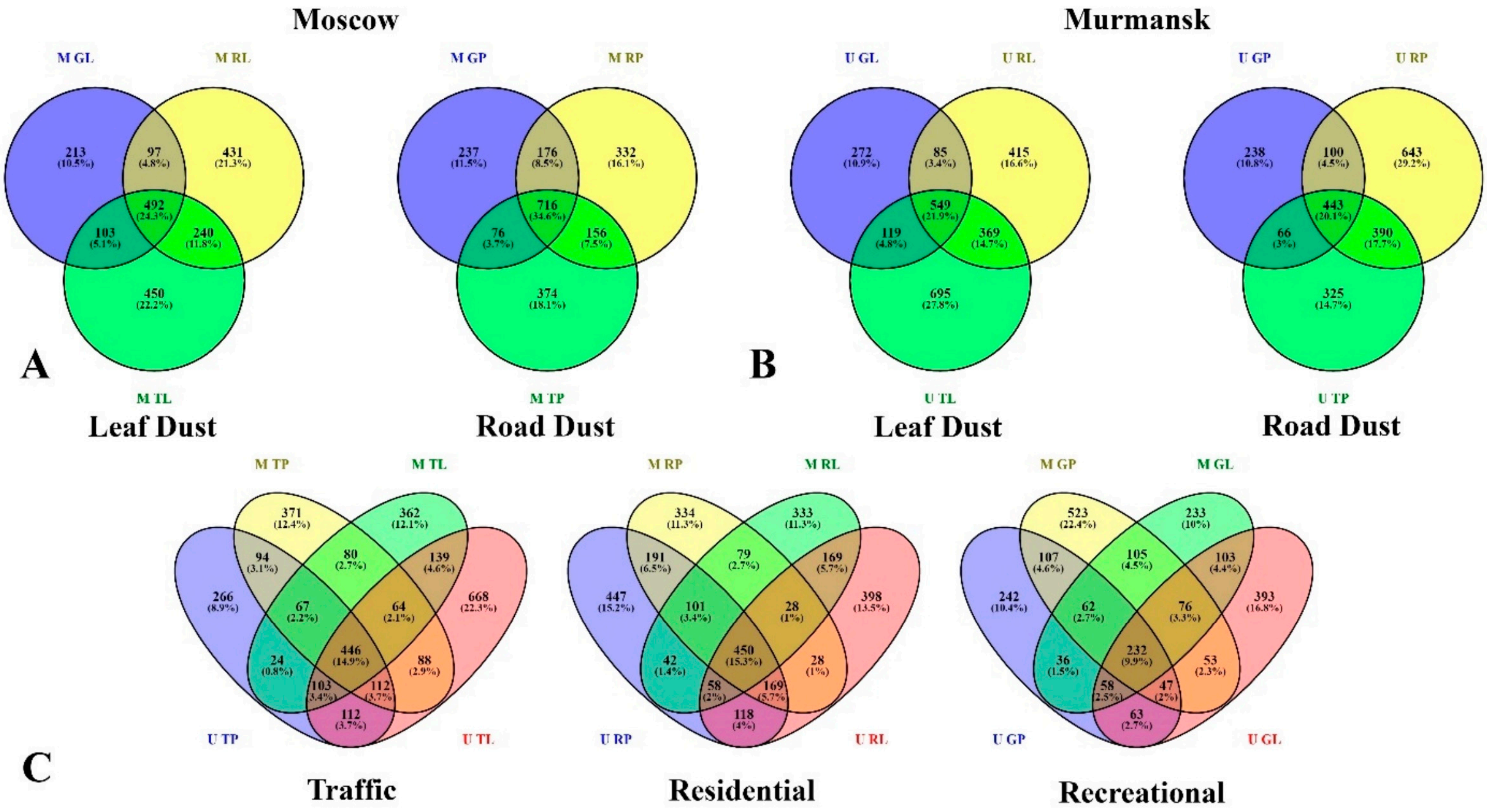

Figure S3. Venn diagrams illustrating the number of unique and shared bacterial OTUs among two biotopes of Moscow (A) and Murmansk(B) detected in three urban sites of cities(C): recreational, residential and traffic zones.

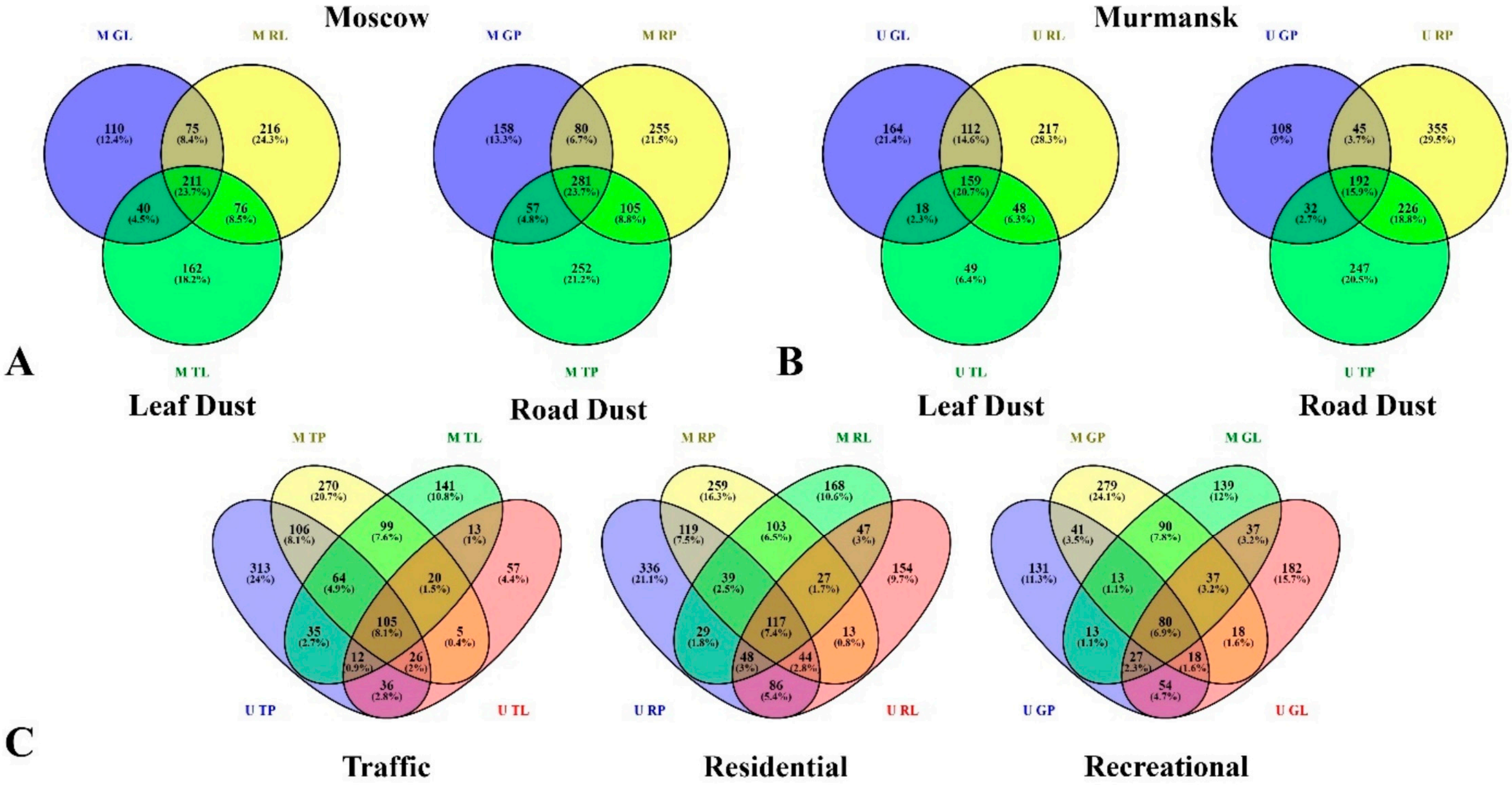

Figure S4. Venn diagrams illustrating the number of unique and shared fungal OTUs among two biotopes of Moscow (A) and Murmansk(B) detected in three urban sites of cities(C): recreational, residential and traffic zones.

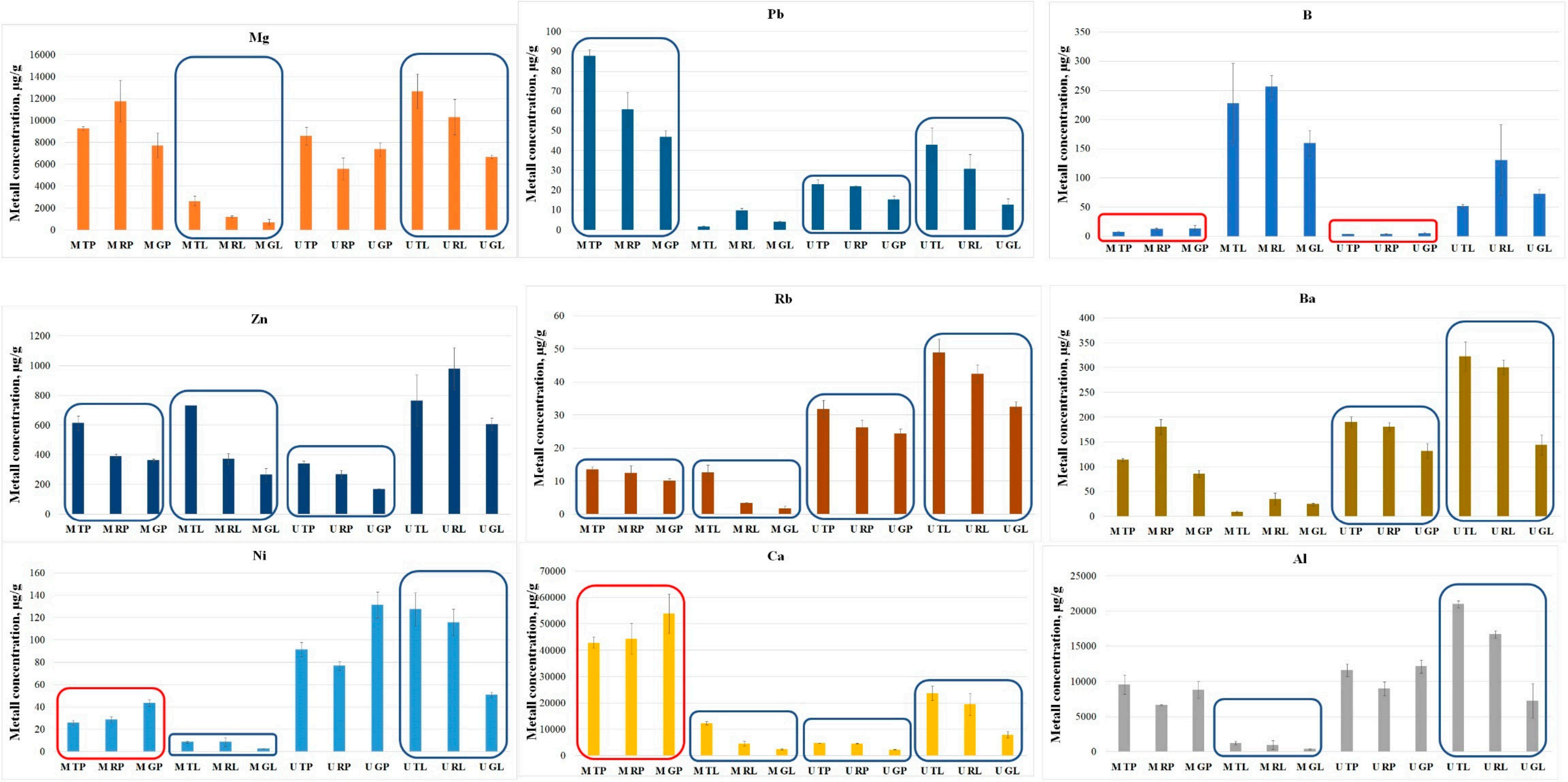

Figure S5. Content of the metal in biotopes of functional zones of Murmansk and Moscow.
M TP, road dust from Moscow traffic zone; M RP, road dust from Moscow residential zone; M GP, road dust from Moscow recreational zone; U TP, road dust from Murmansk traffic zone; U RP, road dust from Murmansk residential zone; U GP, road dust from Murmansk recreational zone; M TL, leaf dust from Moscow traffic zone; M RL, leaf dust from Moscow residential zone; M GL, leaf dust from Moscow recreational zone; U TL, leaf dust from Murmansk traffic zone; U RL, leaf dust from Murmansk residential zone; U GL, leaf dust from Murmansk recreational zone. The red box shows the increase, blue box – decrease.

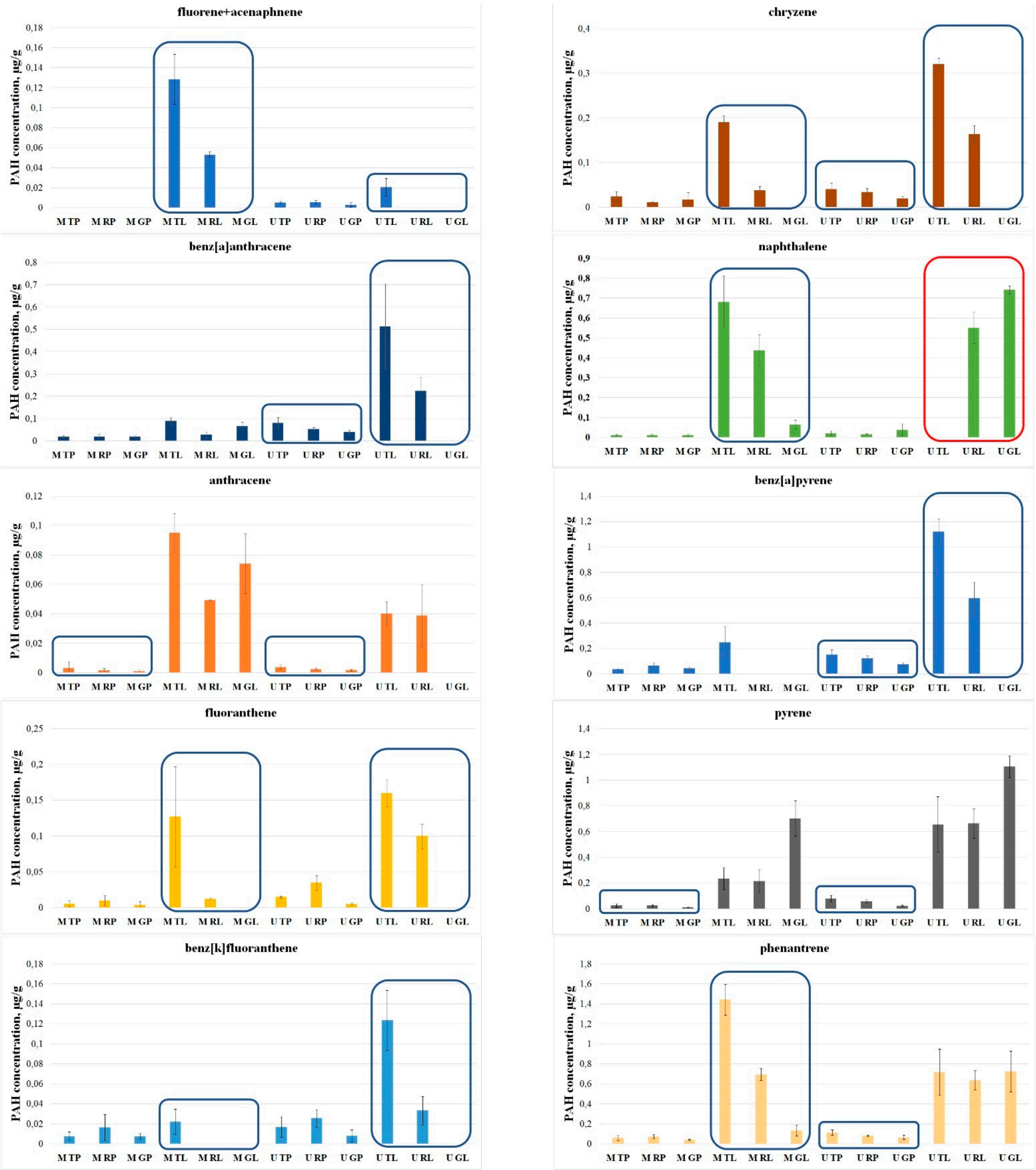

Figure S6. Content of the PAHs in biotopes of functional zones of Murmansk and Moscow.

M TP, road dust from Moscow traffic zone; M RP, road dust from Moscow residential zone; M GP, road dust from Moscow recreational zone; U TP, road dust from Murmansk traffic zone; U RP, road dust from Murmansk residential zone; U GP, road dust from Murmansk recreational zone; M TL, leaf dust from Moscow traffic zone; M RL, leaf dust from Moscow residential zone; M GL, leaf dust from Moscow recreational zone; U TL, leaf dust from Murmansk traffic zone; U RL, leaf dust from Murmansk residential zone; U GL, leaf dust from Murmansk recreational zone. The red box shows the increase, blue box – decrease.

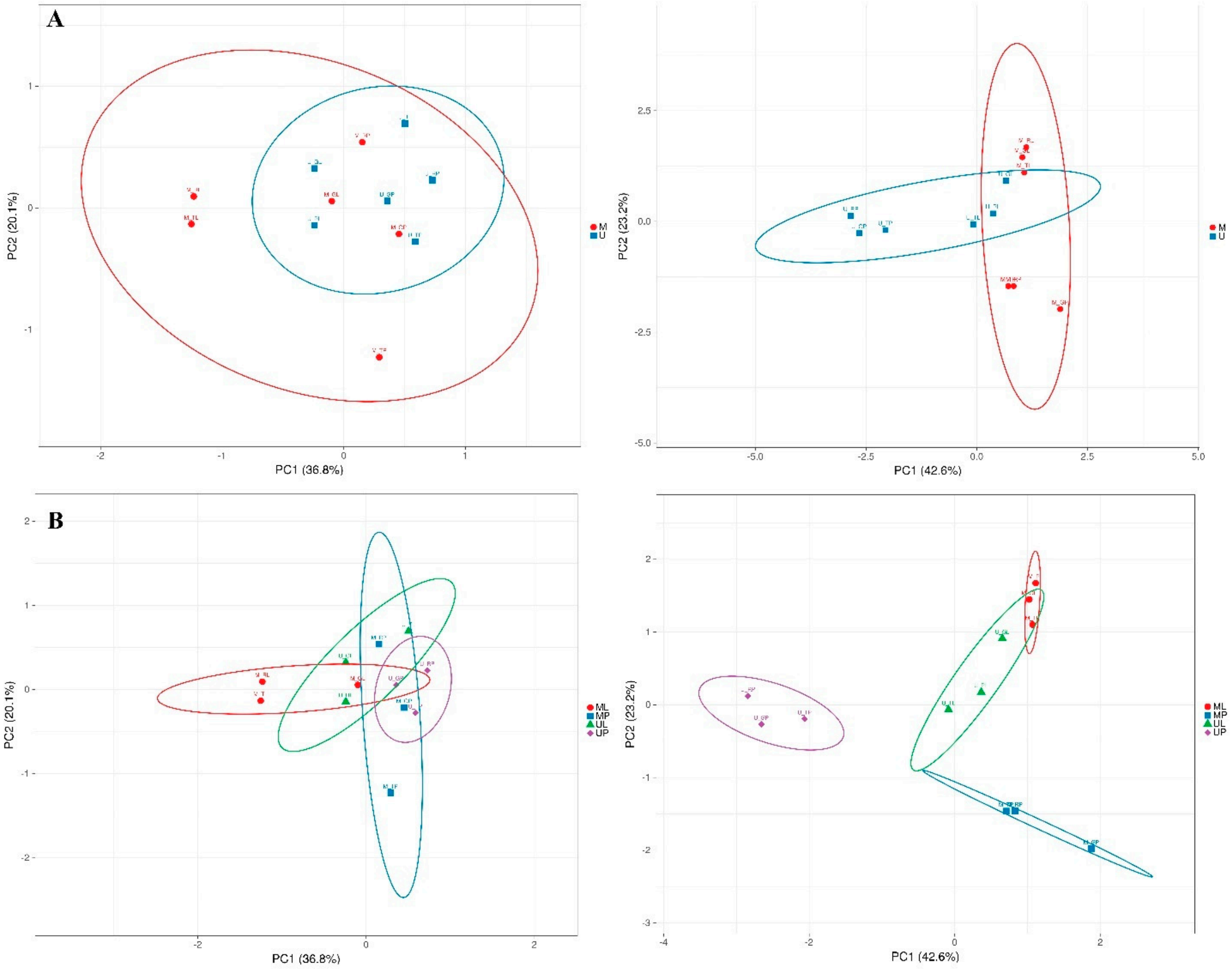

Figure S7. Principal component analysis (PCA) of PAHs (A) and metals (B) composition in biotopes of functional zones of Murmansk and Moscow.

M-Moscow, U – Murmansk, MP – road dust of Moscow, ML – leaf dust of Moscow, UP – road dust of Murmansk, UL – leaf dust of Murmansk.

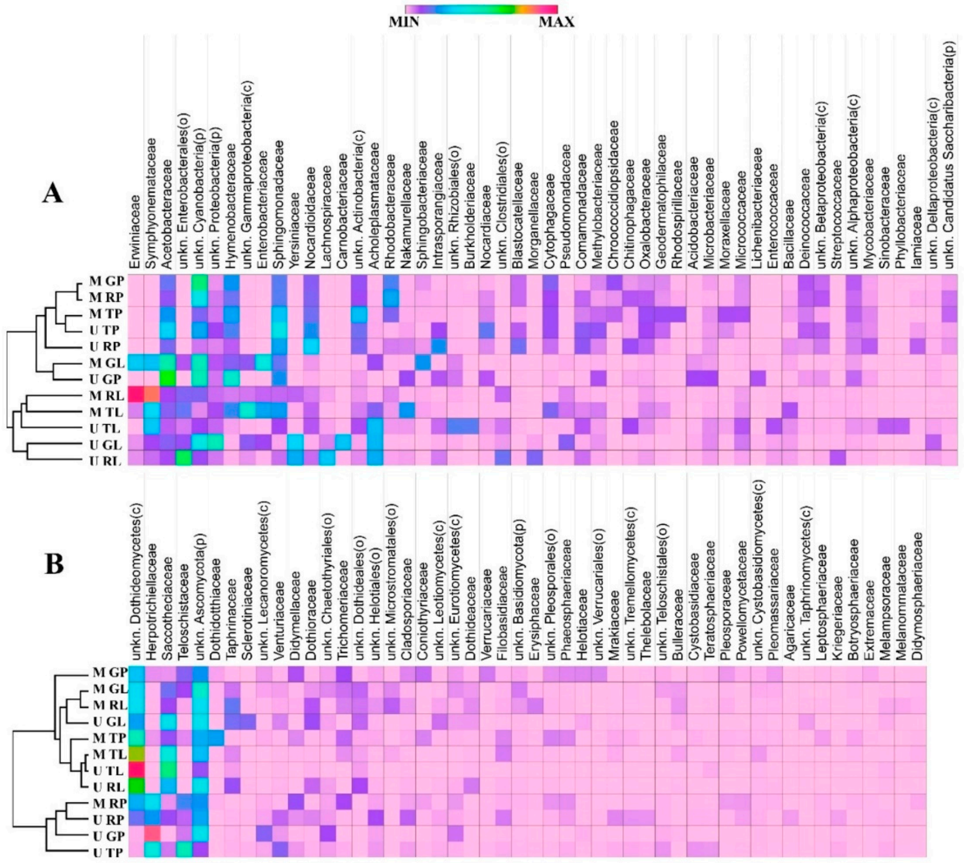

Figure S8. Heat maps of bacterial (A) and fungal (B) species in microbiome of biotopes of functional zones of Murmansk and Moscow.

M TP, road dust from Moscow traffic zone; M RP, road dust from Moscow residential zone; M GP, road dust from Moscow recreational zone; U TP, road dust from Murmansk traffic zone; U RP, road dust from Murmansk residential zone; U GP, road dust from Murmansk recreational zone; M TL, leaf dust from Moscow traffic zone; M RL, leaf dust from Moscow residential zone; M GL, leaf dust from Moscow recreational zone; U TL, leaf dust from Murmansk traffic zone; U RL, leaf dust from Murmansk residential zone; U GL, leaf dust from Murmansk recreational zone. Color code from pink (MIN) to red (MAX) depicts relative amount of respective group.

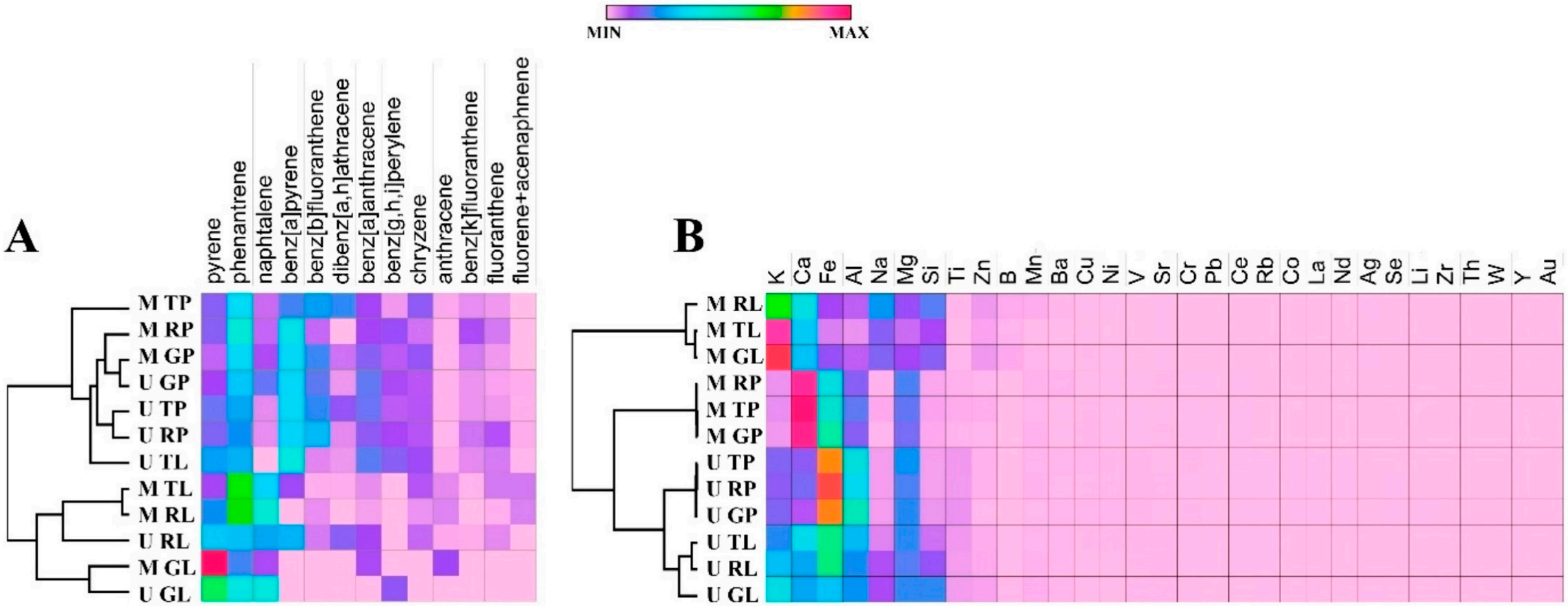

Figure S9. Heat maps of the PAHs (A) and metals (B) in biotopes of functional zones of Murmansk and Moscow.

M TP, road dust from Moscow traffic zone; M RP, road dust from Moscow residential zone; M GP, road dust from Moscow recreational zone; U TP, road dust from Murmansk traffic zone; U RP, road dust from Murmansk residential zone; U GP, road dust from Murmansk recreational zone; M TL, leaf dust from Moscow traffic zone; M RL, leaf dust from Moscow residential zone; M GL, leaf dust from Moscow recreational zone; U TL, leaf dust from Murmansk traffic zone; U RL, leaf dust from Murmansk residential zone; U GL, leaf dust from Murmansk recreational zone. Color code from pink (MIN) to red (MAX) depicts relative amount of respective group.
